# Role of Tomato Lipoxygenase D in Wound-Induced Jasmonate Biosynthesis and Plant Immunity to Insect Herbivores

**DOI:** 10.1371/journal.pgen.1003964

**Published:** 2013-12-12

**Authors:** Liuhua Yan, Qingzhe Zhai, Jianing Wei, Shuyu Li, Bao Wang, Tingting Huang, Minmin Du, Jiaqiang Sun, Le Kang, Chang-Bao Li, Chuanyou Li

**Affiliations:** 1 State Key Laboratory of Plant Genomics, National Centre for Plant Gene Research (Beijing), Institute of Genetics and Developmental Biology, Chinese Academy of Sciences, Beijing, China; 2 State Key Laboratory of Integrated Management of Pest Insects & Rodents, Institute of Zoology, Chinese Academy of Sciences, Beijing, China; 3 Institute of Vegetable, Qingdao Academy of Agricultural Sciences, Qingdao, China; 4 Vegetable Research Center, Beijing Academy of Agriculture and Forestry Sciences, Beijing, China; National University of Singapore and Temasek Life Sciences Laboratory, Singapore

## Abstract

In response to insect attack and mechanical wounding, plants activate the expression of genes involved in various defense-related processes. A fascinating feature of these inducible defenses is their occurrence both locally at the wounding site and systemically in undamaged leaves throughout the plant. Wound-inducible proteinase inhibitors (PIs) in tomato (*Solanum lycopersicum*) provide an attractive model to understand the signal transduction events leading from localized injury to the systemic expression of defense-related genes. Among the identified intercellular molecules in regulating systemic wound response of tomato are the peptide signal systemin and the oxylipin signal jasmonic acid (JA). The systemin/JA signaling pathway provides a unique opportunity to investigate, in a single experimental system, the mechanism by which peptide and oxylipin signals interact to coordinate plant systemic immunity. Here we describe the characterization of the tomato *suppressor of prosystemin-mediated responses8* (*spr8*) mutant, which was isolated as a suppressor of (pro)systemin-mediated signaling. *spr8* plants exhibit a series of JA-dependent immune deficiencies, including the inability to express wound-responsive genes, abnormal development of glandular trichomes, and severely compromised resistance to cotton bollworm (*Helicoverpa armigera*) and *Botrytis cinerea*. Map-based cloning studies demonstrate that the *spr8* mutant phenotype results from a point mutation in the catalytic domain of TomLoxD, a chloroplast-localized lipoxygenase involved in JA biosynthesis. We present evidence that overexpression of *TomLoxD* leads to elevated wound-induced JA biosynthesis, increased expression of wound-responsive genes and, therefore, enhanced resistance to insect herbivory attack and necrotrophic pathogen infection. These results indicate that *TomLoxD* is involved in wound-induced JA biosynthesis and highlight the application potential of this gene for crop protection against insects and pathogens.

## Author Summary

Plants have evolved sophisticated strategies to defend themselves against insect attack. Wound-inducible proteinase inhibitors (PIs) in tomato (*Solanum lycopersicum*) provide an attractive model to understand the signal transduction events leading from localized injury to the systemic expression of defense-related genes. A wealth of evidence indicates that the peptide signal systemin and the phytohormone jasmonic acid (JA) work together in the same signaling pathway to activate the expression of PIs and other defense-related genes. We have been using a genetic approach to dissect the systemin/JA signaling pathway and to discover important genes that can be used for crop protection. Here we report the characterization of the *suppressor of prosystemin-mediated responses8* (*spr8*) mutant, which is defective in wound-induced defense gene expression and therefore is more susceptible to insect attack. We demonstrate that *spr8* defines the *TomLoxD* gene, which encodes a chloroplast-localized lipoxygenase involved in wound-induced JA biosynthesis. Further, we demonstrate that genetic manipulation of *Spr8*/*TomLoxD* leads to increased plant resistance against insect attack and pathogen infection.

## Introduction

Higher plants respond to insect attack and wounding by activating the expression of genes involved in herbivore deterrence, wound healing, and other defense-related processes [1]–[7]. The wound response of tomato (*Solanum lycopersicum*) provides an attractive model to understand the signal transduction events leading from localized injury to the systemic expression of defense-related genes [7], [8]. The principle defensive markers used in these studies are genes encoding proteinase inhibitors (PIs), low molecular weight proteins that inhibit the activity of digestive enzymes in the gut of herbivores [1], [9]. In their milestone study of wound-inducible PIs in tomato, Green and Ryan proposed that specific signals generated at the wound site travel through the plant and activate the expression of PIs and other defense-related genes in remote responding leaves [10].

Systemin, an 18-amino-acid peptide signal, was purified from wounded tomato leaves on the basis of its ability to activate PI accumulation using a convenient bioassay for PI-inducing compounds [9], [11]–[13]. Systemin is derived from the cleavage of a larger precursor protein called prosystemin, which is encoded by a single copy of the *Prosystemin* (*PS*) gene [12], [14]. Transgenic tomato plants that express an antisense *PS* are defective in wound-induced systemic expression of *PI* genes and are more susceptible to insects [14]. Conversely, transgenic tomato plants (called *35S::PS*) that overexpress *PS* constitutively express high levels of PIs without wounding and are more resistant to insects [15], [16]. In addition, genetic analysis in tomato has shown that genes required for (pro)systemin signaling are also essential for wound-induced expression of defensive genes [3], [17], [18]. Together, these genetic studies support that the peptide signal systemin acts as an upstream component of the wound-induced signaling cascades leading to defense gene expression.

It is generally believed that wounding and insect attack lead to the rapid cleavage of systemin from prosystemin. Binding of systemin to its proposed receptor on the cell surface then activates defense gene expression by increasing the endogenous levels of jasmonic acid (JA) and related pentacyclic oxylipins (collectively referred to here as JAs) that are derived from the linolenic acid via the octadecanoid pathway [1], [19]–[21]. A role for JAs in intercellular signaling is supported by the fact that application of MeJA (the methyl ester of JA) to one tomato leaf induces PI expression in distal untreated leaves [22]. JAs are now considered to be key regulators for stress-induced gene expression in virtually all plant species [1], [20], [23]–[27]. It was proposed that systemin and JA work together in the same signal transduction pathway to regulate the systemic expression of defense-related genes [1], [9], [20]. Thus, the systemin/JA signaling pathway for induced resistance in tomato provides a unique opportunity to investigate, in a single experimental system, the mechanism by which peptide and oxylipin signals interact to coordinate systemic expression of defense-related genes [7], [8].

We have been using a genetic approach to dissect the systemin/JA signaling pathway and to elucidate the role of systemin and JA in it. Genetic screen to identify mutations that suppress the constant wound signaling phenotype (i.e., constitutive expression of *PIs* and other defense-related genes) of *35S::PS* plants has led to the identification of several important components of the systemin/JA signaling pathway 17,18,28,29. Significantly, several of the characterized *spr* (*suppressors of prosystemin-mediated responses*) mutants actually define genes that are directly involved in JA biosynthesis or signaling [17], [18], [29]. For example, *Spr2* encodes a chloroplast fatty acid desaturase that catalyzes the ω3 desaturation of linoleic acid (18∶2) to linolenic acid (18∶3), the metabolic precursor for JA biosynthesis [18]. *spr6*, on the other hand, defines the tomato homolog of CORONATINE INSENSITIVE1 (COI1), which has been shown to be the JA receptor in *Arabidopsis* [29], [30]. These studies provided direct evidence that JA acts downstream of systemin in regulating wound-induced expression of defense-related genes.

Grafting experiments conducted with the JA biosynthesis mutant *spr2* and the JA signaling mutant *jai-1* revealed that systemic defense signaling requires both the biosynthesis of JA at the site of wounding and the ability to perceive JA in remote tissues, suggesting that JA acts as a systemic wound signal [3]. Grafting experiments also demonstrated that the graft-transmissible wound signal generated by the *35S::PS* plants can be readily recognized by *spr2* plants (which are insensitive to systemin), but cannot be recognized by the JA-insensitive *jai-1* plants, strongly suggesting that the *35S::PS*-derived wound signal is JA, rather than systemin [3]. These results challenge the previous paradigm that systemin is the long-distance mobile signal for wound-induced defense gene expression [8], [31], [32].

Genetic analyses of tomato wound response also provide insight to understand how the peptide signal systemin interacts with JA to promote systemic defense signaling. In contrast to other tomato wound response mutants that lack both local and systemic PI expression in response to wounding, *spr1* plants were deficient mainly in the systemic response. Moreover, *spr1* abolished JA accumulation in response to exogenous systemin, and showed reduced JA accumulation in wounded leaves [28] Analysis of reciprocal grafts between *spr1* and wild-type (WT) plants showed that *spr1* impedes systemic PI expression by blocking the production of the long-distance wound signal in damaged leaves, rather than inhibiting the recognition of that signal in systemic undamaged leaves. These experiments suggest that *Spr1* is involved in a signaling step that couples systemin perception to the activation of the octadecanoid pathway [28]. These and other studies support that systemin acts locally at the site of wounding to amplify the production of JA, which in turn functions as a mobile signal to activate systemic defense responses [8], [28], [33]. In addition to systemin, the hydroxyproline-rich glycopeptides (HypSys peptides), which are isolated from tomato and tobacco leaves, are also powerful activators of PI expression [34]. Recent genetic data support that, similar to systemin, HypSys peptides also play a role in an amplification loop that upregulates JA production to effect strong systemic defense response [35].

Toward understanding the molecular mechanism of systemin/JA-mediated systemic defense signaling in tomato, we are conducting an enlarged genetic screen to identify more *spr* mutants that suppress the constitutive wound signaling phenotype of the *35S::PS* plants [29]. Here we report the genetic and molecular characterization of *spr8*, a semidominant mutant that is defective in wound-induced expression of defense-related genes. Map-based cloning studies reveal that *Spr8* encodes tomato lipoxygenase D (TomLoxD), a 13-lipoxygenase that catalyzes the hydroperoxidation of linolenic acid, a key step in JA biosynthesis [19]. We show that overexpression of *TomLoxD* leads to elevated wound-induced JA biosynthesis, increased expression of wound-responsive genes and, therefore, enhanced resistance to insects and necrotrophic pathogens. These results highlight the application potential of the *TomLoxD* gene for crop protection.

## Results

### *spr8* Impairs Wound-Induced Expression of Defensive Genes

**Figure 1:**
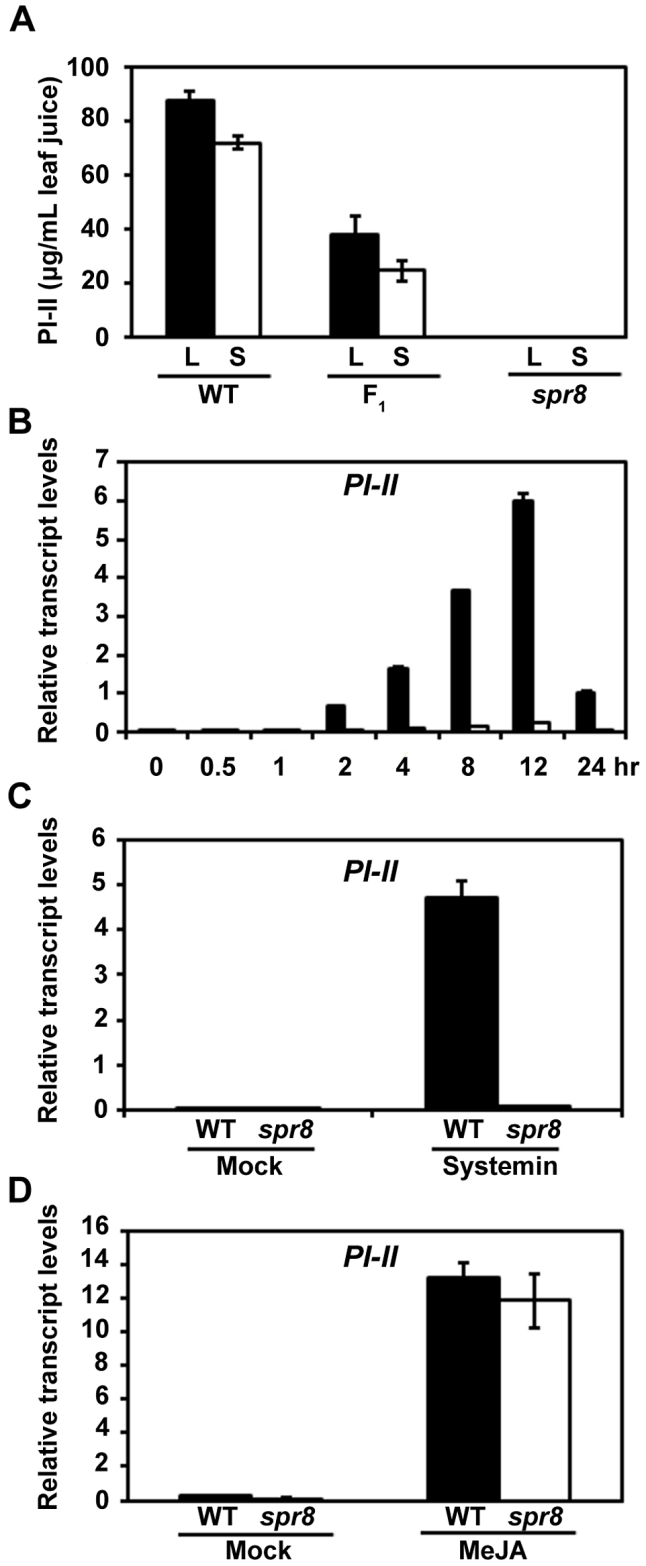
*spr8* impairs the wound-induced expression of PI-II. (A) PI-II protein accumulation in tomato leaves in response to mechanical wounding. Sixteen-day-old wild-type (WT), (WT×*spr8*) F_1_ (F_1_) and *spr8* seedlings were wounded using a hemostat as described in Materials and Methods. Twenty-four hours after wounding, PI-II levels were measured in the wounded leaf (black bar; L, local response) and the upper unwounded leaf (white bar; S, systemic response). Values represent the mean ± SD of six plants. (B) Time-course of wound-induced expression of *PI-II* in WT and *spr8* plants. Sixteen-day-old seedlings of WT (black bar) and *spr8* (white bar) plants containing two fully expanded leaves were mechanically wounded with a hemostat on both leaves for indicated times before total RNAs were extracted for qRT-PCR assays. Data presented are mean values of three biological repeats with SD. (C) Expression of *PI-II* in WT (black bar) and *spr8* (white bar) plants in response to exogenous systemin. Sixteen-day-old seedlings WT and *spr8* seedlings were excised at the base of the stem and supplied with 15 mM sodium phosphate buffer (white bar), or buffer solution with 2.5 pmol systemin. *PI-II* transcription levels were measured 12 h after treatment. Data presented are mean values of three biological repeats with SD. (D) MeJA-induced *PI-II* expression in WT and *spr8* plants. Sixteen-day-old seedlings of WT (black bar) and *spr8* (white bar) plants were treated with MeJA for 12 hours before *PI-II* expression were quantified with qRT-PCR. Data presented are mean values of three biological repeats with SD.

*spr8* is one of the newly identified mutants that can block the constitutively high activity of polyphenol oxidase (PPO) in the *35S::PS* plants [29]. Further characterization of *spr8* was carried out using a *spr8/spr8* homozygous line in which the *35S::PS* transgene was removed by five successive backcrosses to the WT cv. Castlemart (CM). The overall plant morphology, flower development and pollen viability of *spr8* plants were indistinguishable from those of WT plants (Figure S1). The wound response of *spr8* was compared with that of WT using the classical radial immunodiffusion assay for the measurement of wound-induced accumulation of proteinase inhibitor II (PI-II) [11], [29], [36]. For these experiments, 16-day-old seedlings containing two fully expanded leaves were wounded and the accumulation of PI-II protein was quantified. Wounding the lower leaves of WT caused the well-known accumulation of PI-II both in the wounded leaves (local response) and in the upper unwounded leaves (systemic response) (Figure 1A). In contrast, *spr8* seedlings accumulated no detectable PI-II protein in the wounded leaves and the upper unwounded leaves (Figure 1A). Consistent with the PI-II protein data, quantitative real-time PCR (qRT-PCR) assays indicated that the *PI-II* transcripts were expressed very weakly in wounded *spr8* leaves as compared to those in WT leaves (Figure 1B). It has been shown that, similar to the *PI* genes [37], protein products of the tomato wound-responsive genes *threonine deaminase* (*TD*) [16] and *leucine amino peptidase A* (*LapA*) [38] have a direct role in deterring insect performance. Our parallel experiments indicated that the wound-induced expression levels of *TD* (Figure S2A) and *LapA* (Figure S2B) were also largely reduced in *spr8* plants compared to those in WT plants. These results demonstrate that the *spr8* mutation impairs wound-induced expression of defensive genes.

To gain additional insight into the wound response phenotype of *spr8*, we examined the capacity of the mutant to respond to various PI-inducing compounds. As previously reported [28], exogenous application of systemin led to strong expression of *PI-II* transcripts in WT plants (Figure 1C). But *spr8* plants failed to express significant levels of *PI-II* transcripts in response to the same concentrations of systemin (Figure 1C), indicating that *spr8* plants are insensitive to systemin. These results are consistent with the fact that *spr8* was identified as a *suppressor of prosystemin*-*mediated responses*. We then examined the response of *spr8* to the methyl ester of JA, MeJA, which is a potent elicitor of *PI-II* expression in WT plants (Figure 1D). As shown in Figure 1D, exogenous application of MeJA readily restored the *PI-II* expression of *spr8* mutants to levels comparable to those of WT plants. These results led us to classify *spr8* into the group of wounding/systemin-insensitive, but JA-sensitive mutants. It is most likely that the *spr8* mutant defines a signaling step that couples the perception of systemin to activation of the JA pathway.

### *spr8* Affects Glandular Trichome Development

**Figure 2:**
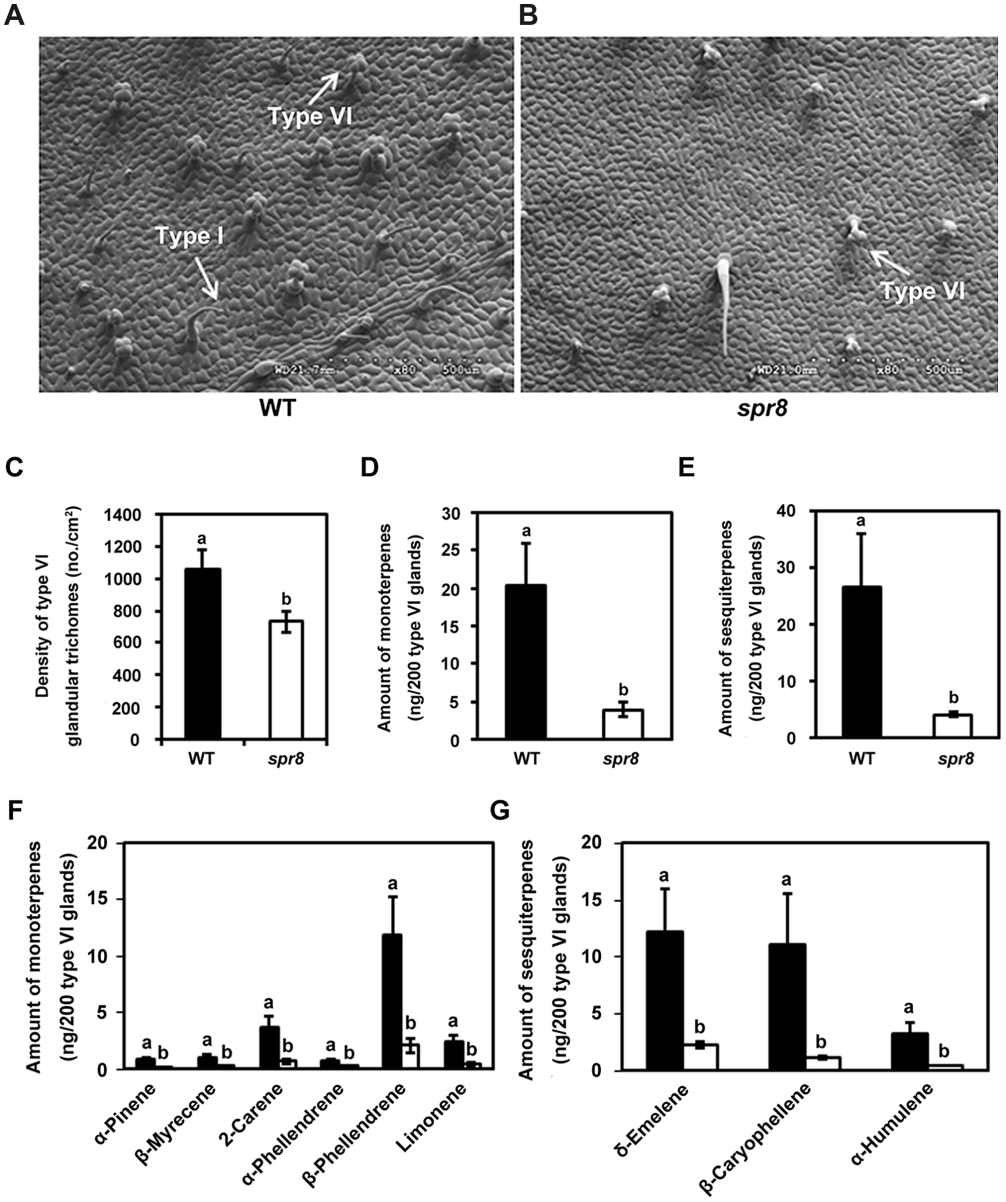
*spr8* impairs trichome development and exhibits defect in type VI glandular trichome exudates. (A) and (B) Scanning electron micrographs of the adaxial surface of a leaflet from WT (A) and *spr8* (B) plants. Five-week-old plants were used for all images. The type I and type VI glandular trichomes were indicated using white arrows, respectively. (C) Mean density (no. per cm^2^ ± SD) of type VI glandular trichomes on the leaflets adaxial surface of WT (black bar) and *spr8* (white bar) plants (n = 10). Samples with the different letters are significantly different at P<0.01 between WT and *spr8*. (D–G) Monoterpene and sesquiterpene content of the type VI glandular trichomes from the adaxial surface of WT (black bar) and *spr8* (white bar) plants leaves. Data presented are mean values of six biological repeats with SD. Samples with the different letters are significantly different at P<0.01 between WT and *spr8*. (D) and (E) Total contents of monoterpene (D) and sesquiterpene (E) of the type VI glandular trichome exudates from WT (black bar) and *spr8* (white bar) leaves. (F) and (G) Comparison of monoterpene (F) and sesquiterpene (G) levels extracted from WT (black bar) and *spr8* (white bar) leaves.

Trichome density and volatile emissions of glandular trichomes provide a formidable protective barrier to invasion by herbivores and pathogens [39]–[41]. Cultivated tomato contains two morphologically distinct types of glandular trichomes. Type I trichomes have an elongated multicellular stalk with a small unicellular vesicle at the tip (Figure 2A and 2B). Type VI trichomes have a unicellular stalk with a four-celled glandular head (Figure 2A and 2B) [42], [43]. In order to determine whether *spr8* affects trichome development, we used scanning electron microscopy to observe the adaxial leaf surface to compare trichome morphology and density between WT and *spr8* plants. A striking feature of *spr8* leaves is the significant reduction of trichome number of both types (Figure 2A and 2B). Quantification of trichomes of five-week-old WT plants (containing at least five leaves) showed that the density of type VI trichome was ∼10 trichomes/mm^2^ on the base region of the third leaflet. Analysis of comparable *spr8* leaflets showed that, type VI trichome density of the mutant was about 70% of that of WT leaflets (Figure 2C).

Next, we used gas chromatography analysis to determine whether *spr8* affects the production of compounds that are synthesized in trichome glands. For these experiments, type VI glandular trichomes were selectively collected by using a stretched-glass pipette and were extracted with methyl tert-butyl ether (MTBE) (see method). Trichome exudates were then analyzed by gas chromatography to measure the terpene composition. From 1, 000 type VI glands collected from the adaxial surface of WT leaves, six monoterpenes (α-pinene, β-myrecene, 2-carene, α-phellandrene, β-phellandrene and limonene; Figure 2F) and three sesquiterpenes (δ-elemene, β-caryophyllene, and α-humulene; Figure 2G) were identified. Comparison of terpene profiles revealed that, all of these compounds were also detected in exudates from the same number of type VI glandular trichomes of *spr8* leaflets, but their accumulation levels were significantly decreased in the mutant (Figure 2F and 2G). In *spr8* glandular trichomes, the accumulation levels of total monoterpenes and sesquiterpenes were reduced to 19.5% and 15.2%, respectively, of those of their WT counterparts (Figure 2D and 2E). These results support the hypothesis that the *spr8* mutation affects the terpene metabolic pathway that mainly operates in type VI trichome glands.

### *spr8* Plants Are Compromised in Resistance against Chewing Insects

**Figure 3:**
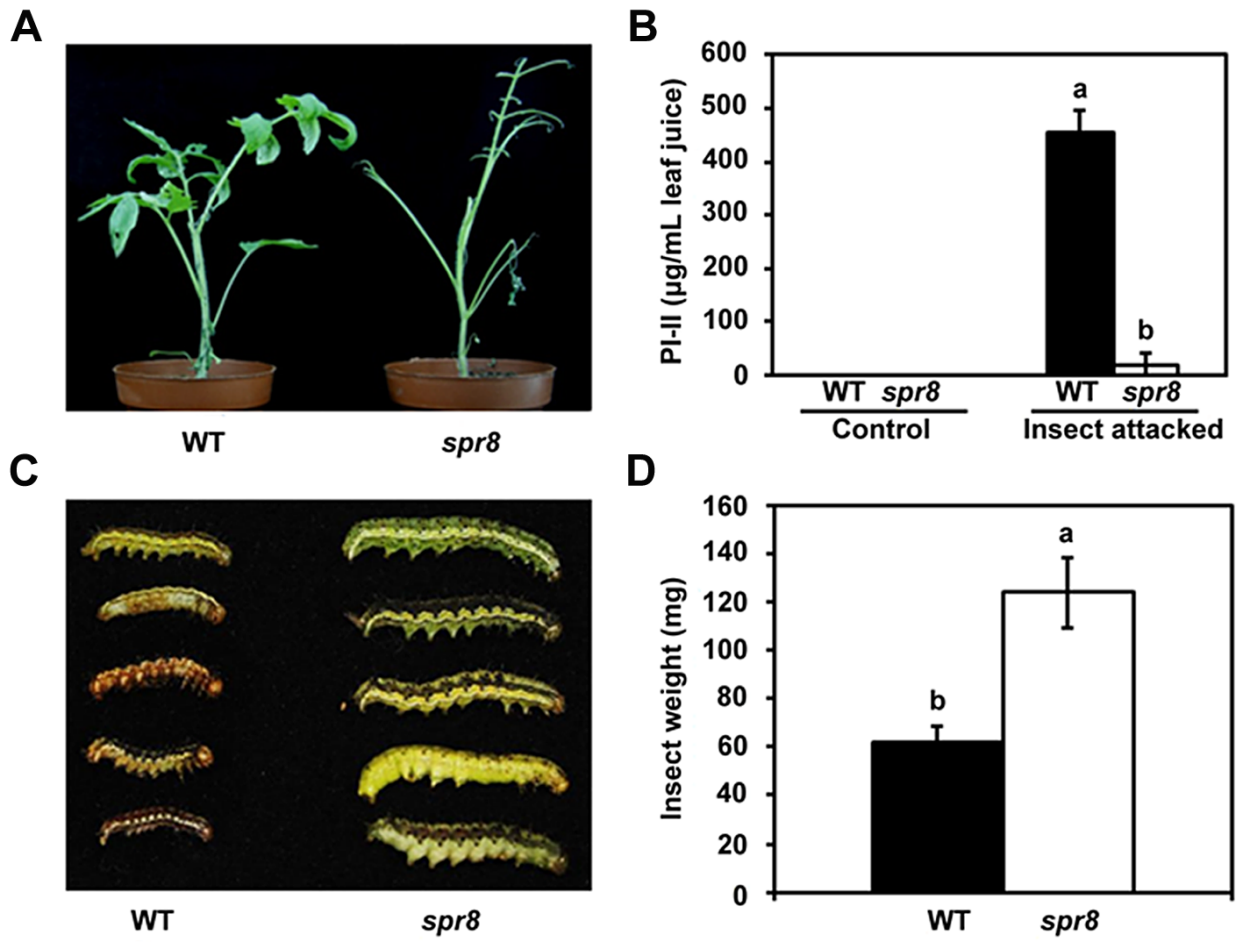
*spr8* plants show reduced resistance to cotton bollworm larvae (*Helicoverpa armigera*). (A) Representative WT (left) and *spr8* (right) plants at the end of cotton bollworm larvae feeding trial. (B) PI-II proteins accumulation in WT (black bar) and *spr8* (white bar) leaves in response to cotton bollworm larvae feeding (n = 5). (C) Size of larvae recovered at the end of cotton bollworm feeding trial. (D) Larval weight recovered at the end of 7 d of feeding trial on whole plants of WT (black bar) and *spr8* (white bar) (n = 15). In (B) and (D), data shown are the mean with SD. Bars with different letters are significantly different from each other (P = 0.05). The feeding trails on whole plants were repeated three times with similar results. In each experiment, 10 newly hatched larvae were placed on at least six five-week-old plants of each genotype. Larvae were allowed to feed on the same plant for the duration of the trial.

The inability of *spr8* plants to express significant levels of defensive genes in response to mechanical wounding and systemin (Figure 1 and Figure S2) suggests that this mutant is compromised in resistance to herbivorous insects. To test this hypothesis, newly hatched cotton bollworm (*Helicoverpa armigera*) larvae were placed on leaves of 5-week-old plants to initiate a feeding trial. Sustaining long-term feeding by insects, *spr8* plants were severely damaged (Figure 3A, right), while WT plants showed relatively few signs of macroscopic damage during the period of the feeding trial (Figure 3A, left). After termination of the feeding trial, PI-II protein accumulation in the remaining leaf tissues was measured, as was the weight gain of larvae reared on both of the host genotypes. In contrast with high levels of PI-II accumulation in herbivore-damaged WT leaves, very little or no PI-II protein accumulation was detected in hornworm-challenged *spr8* plants (Figure 3B). These results indicate that WT plants have relatively high levels of natural resistance to the cotton bollworm larvae and that this resistance is severely compromised by the *spr8* mutation. Consistently, the average weight of larvae reared on the mutant was 2.0-fold greater than that of larvae reared on WT plants (Figure 3C and 3D). These results demonstrate that *Spr8* is required for the resistance of tomato plants to attacking hornworm larvae.

### The Wound-Response Phenotype of *spr8* Results from a Defect in the *TomLoxD* Gene

**Figure 4:**
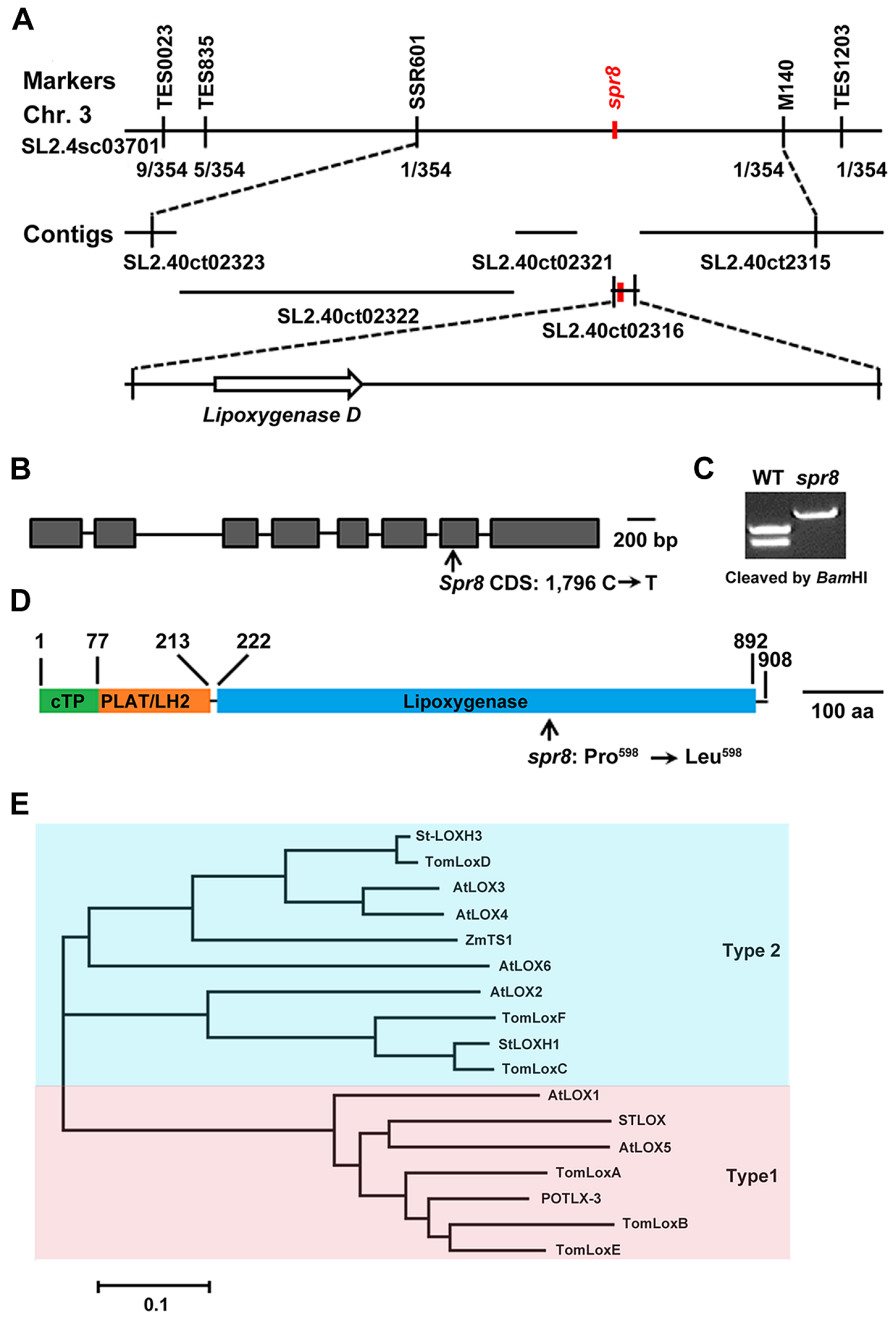
Map-based cloning of *Spr8*. (A) Fine genetic and physical mapping of *Spr8*. Numbers below the line indicate the number of recombination events identified between markers. Placement of *Spr8* on SL2.40ct02316 was determined by the phenotypic data. (B) Gene structure of *Spr8/TomLoxD*. Introns and exons are indicated by horizontal lines and closed boxes, respectively, and are drawn to scale. Arrow indicates the point mutation site of *spr8*, which is the *Bam*HI recognition site GGATCC. Bar = 100 bp. (C) Development of a CAPS marker to distinguish *spr8* mutants from WT plants. Parts of the *TomLoxD* gene were amplified from genomic DNAs of both WT and *spr8* alleles using the PCR primer pair P1 (5′-TTTCCAATGTCAGTATATAACTC-3′) and P2 (5′-CCATTTCTCGATCGGATCAATG -3′). *Bam*HI cleaved the 680 bp DNA fragment at the recognition site GGATCC in 229 bp and 451 bp, but not from the *spr8* mutant, in which the recognition site was altered to GGATCT by the mutation. (D) The TomLoxD protein contains a predicted chloroplast transit peptide (cTP, green), the PLAT/LH2 beta-barrel (orange), and the lipoxygenase domain (blue). Arrow indicates the point mutation site of Spr8 protein, at which Pro^598^ changes to Leu^598^. Bar = 100 amino acid (aa). (E) Phylogenetic tree of various lipoxygenases from tomato, potato, *Arabidopsis* and maize. Shown is the sequence relatedness between the deduced amino acid sequence of TomLoxD and other lipoxygenases.

Genetic analysis revealed that *spr8* is a semi-dominant mutant, given that the wound-response phenotype of the heterozygous (*Spr8/spr8*) plants was intermediate between those of the homozygous *spr8* plants and their WT counterparts (Figure 1A and Figure S3). The deficiency in wound-induced PI-II protein accumulation of *spr8* provides a facile assay for map-based cloning studies to determine the genetic basis of this defect. A combination of cleaved amplified polymorphic sequence (CAPS) and simple sequence repeat (SSR) markers was used to localize *Spr8* to a region on the long arm of chromosome 3 between SSR markers TES0023 and TES1203 (Figure 4A). Fine mapping using 354 backcrossed (BC_1_) individuals showing a WT wound response delimited the *Spr8* locus to a region between the markers SSR601 and M140 in the scaffold SL2.40sc03701 of the sequenced tomato genome [44], [45]. Among the genes predicted by the International Tomato Annotation Group (ITAG2.3 release, http://solgenomics.net) in this interval, *Solyc03g122340*, which encodes TomLoxD (tomato lipoxygenase D), a wound-inducible lipoxygenase [46], is considered to be a strong candidate of *Spr8*. DNA sequencing revealed that *spr8*-derived *TomLoxD* complementary DNA (cDNA) contains a single C-to-T mutation (Figure 4B). This C-to-T mutation, which was confirmed by sequencing of PCR-amplified genomic DNA from *spr8* plants, destroys a *Bam*HI restriction site, and a CAPS marker was developed to detect the *spr8* mutant allele (Figure 4C). The single base pair change in the *TomLoxD* gene is predicted to replace a highly conserved (i.e., invariant among plant and animal lipoxygenases) Pro residue at position 598 with an Leu (Figure 4D and Figure S4).

Considering that *spr8* is a semi-dominant mutation, we performed the following experiments to show that the missense mutation in *TomLoxD* accounts for the wound response phenotype of *spr8*. First, transgenic plants overexpressing a WT allele of *TomLoxD* (*TomLoxD-OE*) showed increased wound response in term of wound-induced defense gene expression (See below). Second, similar to *spr8* plants, transgenic plants expressing a *TomLoxD* RNA interference (RNAi) construct (*TomLoxD-RNAi*) were defective in wound-induced expression of *PI-II* (Figure S5A and S5B). Third, the wound response phenotype of transgenic plants overexpressing a mutant allele of *TomLoxD* (*TomLoxD^P598L^-OE*) was intermediate between that of the homozygous *spr8* plants and their WT counterparts (Figure S5A and S5B). Finally, overexpression of a WT allele of *TomLoxD* in the *spr8* background failed to fully rescue the wound response defects of the mutant (Figure S5C and S5D). Collectively, these results support that the identified C-to-T mutation in the *TomLoxD* gene is responsible for the wound response phenotype of *spr8* plants and that the *spr8* allele of *TomLoxD* (i.e., *TomLoxD^P598L^*) acts as a dominant negative regulator of the tomato wound response pathway.

Lipoxygenases are nonheme iron-containing fatty acid dioxygenases that catalyze the peroxidation of polyunsaturated fatty acids such as linoleic acid, α-linolenic acid, and arachidonic acid [47]. Based on the positional specificity of linoleic acid oxygenation, they are classified as 9-lipoxygenases (oxygenation occurs at carbon 9 of the hydrocarbon backbone) and 13-lipoxygenases (oxygenation occurs at carbon 13 of the hydrocarbon backbone). 13-lipoxygenases can be further divided as types 1 and 2 based on the presence of a putative chloroplast transit peptide (cTP) [47]. ChloroP (http://www.cbs.dtu.dk/services/TargetP/)-based analysis predicted that the deduced amino acid sequence of TomLoxD contains a putative cTP (TomLoxD^1–77^), a small N-terminal PLAT/LH2 domain (TomLoxD^78–213^) that forms a β-barrel, and a C-terminal domain (TomLoxD^222–892^) that forms α-helices (Figure 4D). It is generally believed that the N-terminal β-barrel domain is involved in membrane or substrate binding, whereas the C-terminal domain harbors the catalytic site of the enzyme [48]. This primary protein structure suggests that TomLoxD is a member of the type 2 plastid-localized 13-lipoxygenases [47]. This prediction is supported by our phylogenetic analysis of plant lipoxygenases, which places TomLoxD in a clade including functionally characterized and predicted type 2 13-lipoxygenases (Figure 4E). To confirm the chloroplast localization of the TomLoxD protein, full-length of the *TomLoxD* cDNA was fused to the green fluorescent protein (GFP) reporter gene and subsequently transformed into *Arabidopsis* leaf protoplast cells. As shown in Figure S6, the GFP fluorescence was co-localized with the red chlorophyll autofluorescence, suggesting that TomLoxD is a chloroplast-localized protein. Notably, in our phylogenetic analysis, TomLoxD was most similar to the *Arabidopsis* LOX3 and LOX4 (71.7% and 71.3% amino acid identity, respectively) (Figure 4E), which has recently been shown to be type 2 chloroplast-localized 13-lipoxygenases that are involved in JA biosynthesis [49]. It is noteworthy that the TomLoxD^P598L^ mutation in *spr8* occurs in the C-terminal α-helices domain, presumably impairs the catalytic activity of the enzyme (Figure 4D).

### The *spr8* Mutation Impairs Wound-Induced JA Biosynthesis

**Figure 5:**
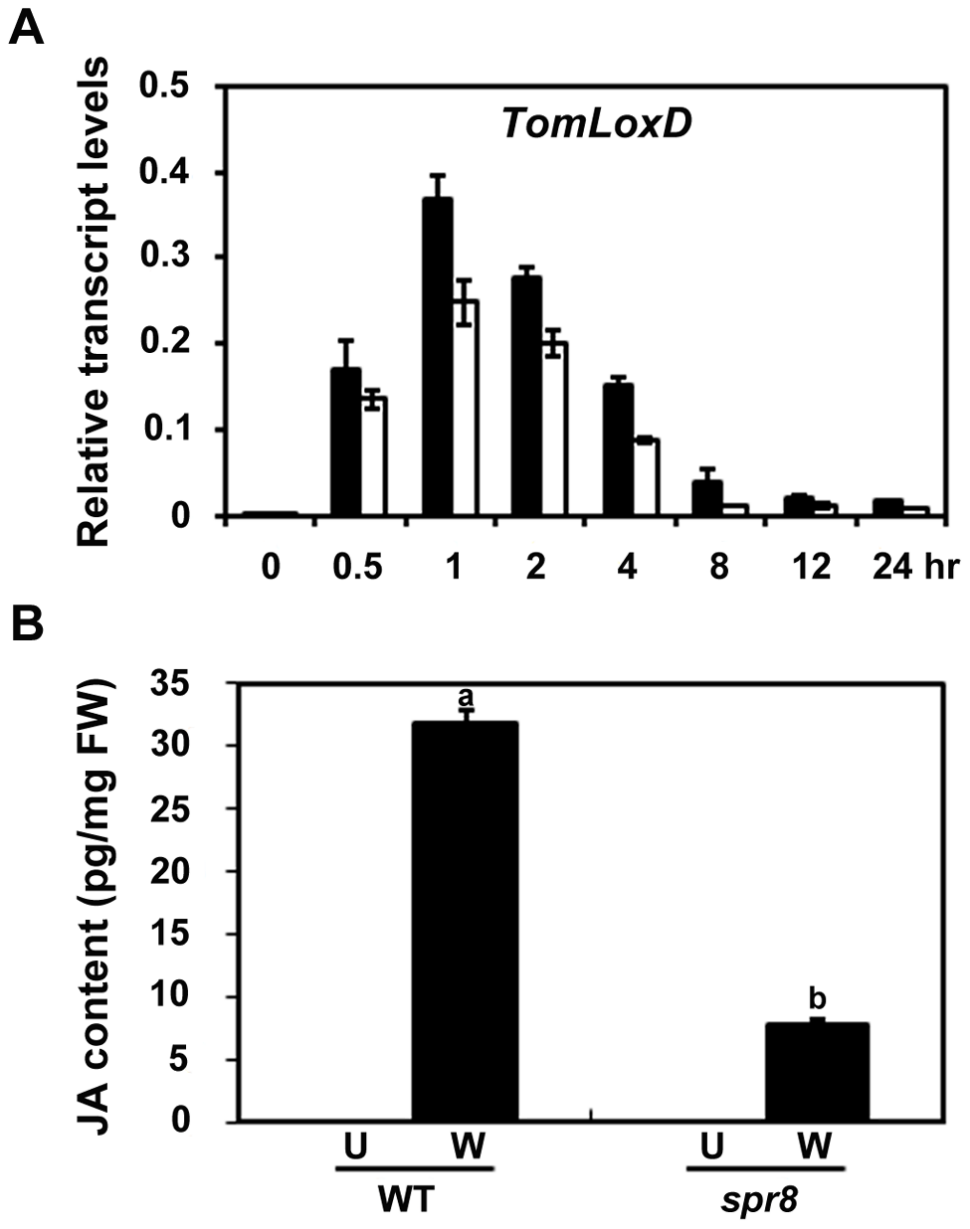
*spr8* impairs wound-induced JA biosynthesis. (A) Time-course transcript levels of *TomLoxD* in response to mechanical wounding. Sixteen-day-old seedlings of WT (black bar) and *spr8* (white bar) plants were mechanically wounded for indicated times before total RNAs were extracted for qRT-PCR assays. Data presented are mean values of three biological repeats with SD. (B) JA levels in response to wounding. WT and *spr8* plants (16-day-old) were mechanically wounded as described above, and JA levels were measured 1 h after wounding (W, Wounded; black bar). JA was also extracted from leaves of unwounded plants (U, Unwounded; white bar). Data show the mean ± SD of three independent samples and are indicative of three independent experiments. Bars with different letters are significantly different compared *spr8* mutant plants with WT plants (P = 0.01). FW, fresh weight.

The above-described results point to a possibility that TomLoxD is a functional 13-lipoxygenase involved in wound-induced JA biosynthesis and that the *spr8* allele of *TomLoxD* (hereafter referred to as *TomLoxD^P598L^*) impairs wound-induced JA biosynthesis. As the first step to prove this, we examined the expression of *TomLoxD* or *TomLoxD^P598L^* in response to wounding. Consistent with a previous investigation [46], the levels of *TomLoxD* transcripts were induced by wounding within 30 min and peaked at 1 h after wounding, *TomLoxD* transcripts then showed a tendency of decline and returned to control levels within 8 h (Figure 5A), indicating that *TomLoxD* is an early wound-inducible gene. Interestingly, the wound-induced expression kinetics of *TomLoxD^P598L^* was essentially similar to that of *TomLoxD*, albeit its expression levels were somehow reduced as compared to that of the latter (Figure 5A). These results indicate that *TomLoxD^P598L^* is still responsive to wounding.

To determine the contribution of TomLoxD and TomLoxD^P598L^ in wound-induced JA biosynthesis, we used liquid chromatography coupled with tandem mass spectrometry (LC-MS/MS) to measure endogenous JA levels in WT and *spr8* plants in response to wounding. We consistently observed that the JA levels in unwounded WT and mutant leaves were below the detection limit (Figure 5B). One hour after wounding, the average JA level was increased to 31.7±1.1 pg per milligram of fresh weight (pg/mg FW) in WT leaves, whereas the average JA level in mutant leaves was only 7.9±0.3 pg/mg FW (P<0.0001, Student's t test) (Figure 5B), confirming that *spr8* plants are defective in wound-induced JA biosynthesis. These results indicate that TomLoxD is required for wound-induced JA biosynthesis and that the TomLoxD^P598L^ mutant allele largely impairs this capability.

Taken together, our data support that, even though the expression of *TomLoxD^P598L^* is still responsive to mechanical wounding (Figure 5A), this mutant version of TomLoxD impairs wound-induced JA biosynthesis (Figure 5B).

### The Wound-Induced Expression of *TomLoxD* Is Directly Regulated by the MYC Transcription Factor SlMYC2

**Figure 6:**
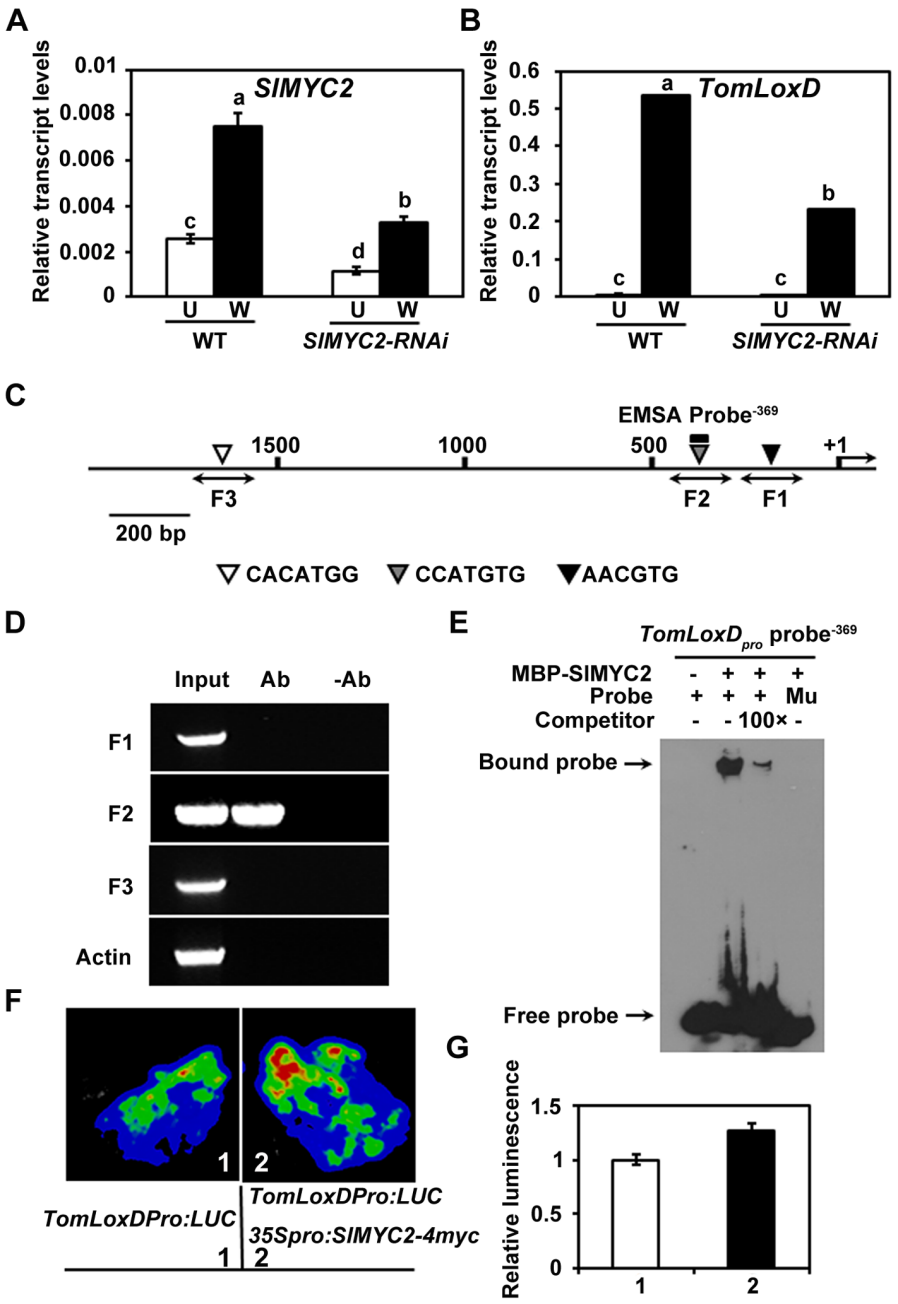
SlMYC2 regulates *TomLoxD* expression through a direct association with its promoter. (A) and (B) Expression of *SlMYC2* (A) and *TomLoxD* (B) in response to wounding. Sixteen-day-old seedlings of WT and *SlMYC2-RNAi* plants were mechanically wounded. Total RNAs were extracted 1 hour after wounding (W, Wounded; black bar) for qRT-PCR. RNAs were also extracted from leaves of unwounded plants (U, Unwounded; white bar) as control. Data presented are mean values of three biological repeats with SD. Data sets marked with different letters are significantly different from each other as assessed by Student's *t* test at *P*<0.001. (C) Schematic diagram of the promoter region of *TomLoxD*. Black lines represent the *TomLoxD* promoter region, including potential SlMYC2 binding G-box-like motif (black, gray and white triangles), DNA fragments (F1, F2 and F3) used for ChIP-PCR, and probe used for EMSA. The translational start sites (ATG) is shown as +1. Bar = 200 bp. (D) Enrichment of the DNA fragment F2 following ChIP using anti-myc antibody. *35S_pro_:SlMYC2-4myc* transgenic seedlings and anti-myc antibody (Millipore) were used in ChIP assays. 16-day-old *35S_pro_:SlMYC2-4myc* plants were mechanically wounded on both leaves, one hour after wounding, leaf tissues were harvested for crosslinking. The “no antibody” (-Ab) immunoprecipitates serve as negative controls. Three biological replicates were performed with similar results. (E) EMSA showing that the MBP-SlMYC2 fusion protein binds to the *TomLoxD_pro_* probe^−369^ of *TomLoxD in vitro*. Biotin-labeled probes were incubated with MBP-SlMYC2 purified proteins, and the free and bound probes were separated in an acrylamide gel. As indicated, unlabeled probes were used as competitors. Similar results were obtained in three independent experiments. Mu, mutated labeled probe in which the G-box motif was deleted. (F–G) Transient expression assays showing that SlMYC2 activates the expression of *TomLoxD*. Representative images of *N. benthamiana* leaves 72 h after infiltration are shown. The bottom panel indicates the infiltrated constructs. (G) Quantitative analysis of luminescence intensity in (F). Values are mean ± SD of five independent determinations.

In the model plant of *Arabidopsis*, much of our understanding of the JA signaling has come from the recent elucidation of the molecular details of JA-regulated gene transcription through the basic helix-loop-helix (bHLH)-type transcription factor MYC2, a master regulator of JA responses [50]–[54]. Considering that in *Arabidopsis* MYC2 directly regulates the expression of several JA biosynthetic genes including *LOX2* [55], it is reasonable to speculate that SlMYC2, the tomato homolog of MYC2, may directly regulate the expression of *TomLoxD*. Indeed, several lines of evidence lends support to this hypothesis. First, wound-induced expression levels of *TomLoxD* were substantially reduced in *SlMYC2-RNAi* plants as compared to those in WT plants (Figure 6A, 6B and Figure S7), indicating that SlMYC2 positively regulates the wound-induced expression of *TomLoxD*; Second, chromatin immunoprecipitation (ChIP) assays using *35S_pro_:SlMYC2-4myc* plants indicated that SlMYC2 associates with a G-box-like motif (CCATGTG) in the promoter region of *TomLoxD* (Figure 6C and 6D); Third, DNA electrophoretic mobility shift assays (EMSA) indicated that a maltose binding protein (MBP)-SlMYC2 fusion protein binds the promoter of *TomLoxD* in a G-box-like motif-dependent manner (Figure 6E). Finally, using the transient expression assay of *Nicotiana benthamiana* leaves, we verified the activation effect of SlMYC2 on the expression of a reporter containing the *TomLoxD* promoter fused with the firefly luciferase gene (LUC) (Figure 6F and 6G). Together, these data demonstrate that the wound-induced expression of *TomLoxD* is under the direct regulation of SlMYC2.

### Overexpression of *TomLoxD* Leads to Increased Plant Immunity to Insects and Necrotrophic Pathogens

**Figure 7:**
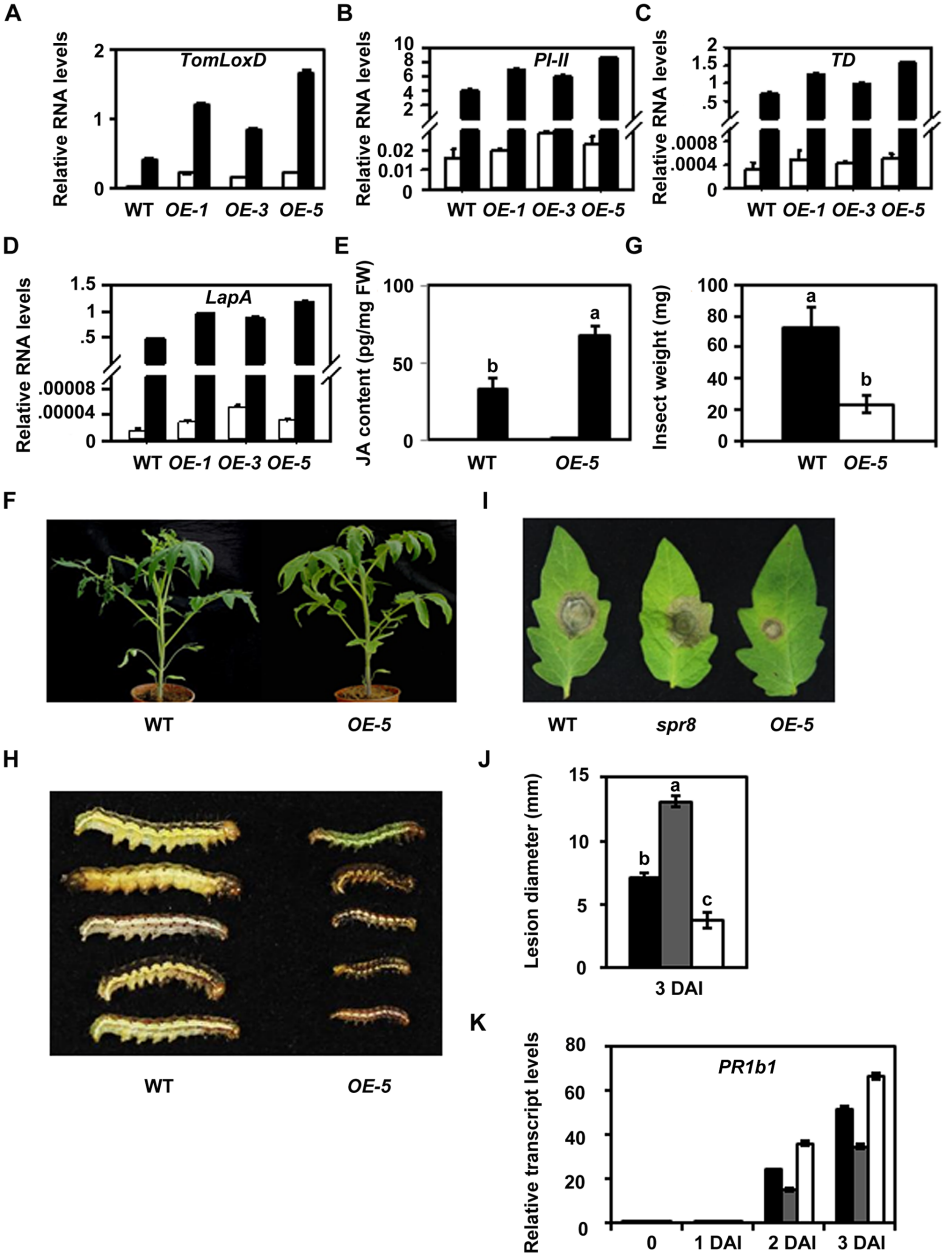
Resistance of *TomLoxD* overexpression plants to cotton bollworm larvae and *Botrytis cinerea*. (A–D) Expression of *TomLoxD* (A), *PI-II* (B), *TD* (C) and *LapA* (D) in *TomLoxD* overexpression plants in response to wounding. Two-leaf-stage plants of WT and *TomLoxD* overexpression lines (*OE-1*, *OE-3* and *OE-5*) were mechanically wounded (black bar). After 1 hour and 12 hours, leaf tissues were harvested for RNA extraction and gene expression analysis. Unwounded leaves (white bar) of each genotype were used as control. Data show the mean ± SD of three independent sample. (E) JA levels in unwounded (U, Unwounded; white bar) and wounded 1 hour (W, Wounded; black bar) leaves of two-leaf-stage WT and *OE-5* plants in response to wounding. Data show the mean ± SD of three independent sample preparations. Bars with different letters are significantly different compared *OE-5* plants with WT plants (P = 0.01). FW, fresh weight. (F–H) *OE-5* plants show increased resistance to insects attack. (F) Representative WT (left) and *OE-5* (right) plants at the end of cotton bollworm larvae feeding trial. (G) Larval weight recovered at the end of the 14-day-feeding trial on whole plants of WT (black bar) and *OE-5* (white bar) (n = 15). Data represent the mean with SD. Bars with different letters are significantly different from each other (P = 0.05). (H) Size of larvae recovered at the end of cotton bollworm feeding trial. The feeding trails on whole plants were performed as described above and were repeated three times with similar results. (I–K) *OE-5* plants exhibit increased resistance to *B. cinerea*. (I) and (J) Detached leaves from five-week-old WT (left), *spr8* (middle) and *OE-5* (right) plants were inoculated with *B. cinerea*. Photograph was taken (I) and the disease lesion diameter analyzed in *Botrytis*-inoculated leaves of WT (black bar), *spr8* (gray bar) and *OE-5* (white bar) at 3 DAI (J). Error bars represent the SD from three independent experiments (n = 30). Data sets marked with different letters are significantly different from each other as assessed by Student's *t* test at P<0.001. (K) Expression of *PR1b1* in response to *B. cinerea* infection. Sixteen-day-old seedlings of WT (black bar), *spr8* (gray bar) and *OE-5* (white bar) were inoculated as described in Material and Method. At different times as indicated, samples were harvested for RNA extraction and qRT-PCR analysis. Data presented are mean values of three biological repeats with SD.

Our findings that *TomLoxD* is required for wound-induced JA biosynthesis and defense gene expression raised the possibility that overexpression of this gene could enhance wound-induced JA biosynthesis, which, in turn, leads to increased plant resistance. To test this hypothesis, we generated transgenic tomato plants overexpressing the *TomLoxD* cDNA driven by the cauliflower mosaic virus 35S promoter (*OE* plants). Increased expression of *TomLoxD* in transgenic lines including *OE-1*, *OE-3* and *OE-5* was confirmed by qRT-PCR analysis (Figure 7A). Under normal growth conditions, the overall growth and morphology of these *OE* plants was essentially similar to those of WT plants (Figure S1). We then compared the expression levels of defensive genes between these *OE* plants and WT plants. Similar steady-state levels of *PI-II*, *TD* and *LapA* transcripts were detected between the noninduced *OE* plants and WT plants (Figure 7B–7D). A marked increase in the accumulation levels of these transcripts was, however, observed in the *TomLoxD* overexpression plants in response to mechanical wounding (Figure 7B–7D). These results demonstrate that overexpression of *TomLoxD* leads to enhanced wound-induced activation of *PI-II* and other defense-related genes.

To test that the increased wound-induced defense gene expression in these *OE* lines may be resulted from enhanced wound-induced accumulation levels of JA, we examined wound-induced JA accumulation between *OE-5* and WT plants. Similar steady-state levels of JA were detected between *OE-5* and WT plants (Figure 7E), indicating that overexpression of *TomLoxD* does not lead to constant accumulation of high levels of JA. In response to mechanical wounding, however, a substantial increase in the accumulation of JA was observed in *OE-5* plants (Figure 7E), indicating that overexpression of *TomLoxD* leads to enhanced wound-induced accumulation of the defense hormone JA.

The ability of *TomLoxD* overexpresser lines to accumulate higher levels of JA and to express increased levels of defensive genes in response to mechanical wounding suggested that these transgenic plants may be more resistant to herbivorous insects. To test this possibility, five-week-old *OE-5* and WT plants were challenged with *Helicoverpa armigera* larvae. After termination of the feeding trial, we examined the weight of the larvae to assess the resistance of plants. The average weight of larvae reared on *OE-5* plants was only 32.5% of that of larvae reared on WT plants (Figure 6F–6H), demonstrating that overexpression of *TomLoxD* leads to enhanced plant resistance to herbivorous insects.

Considering that the JA-signaled plant resistance is also effective to the necrotrophic pathogen *Botrytis cinerea* [50], [51], [56]–[58], we examined the performance of *OE-5* plants to the Hy2-1 strain of *B. cinerea*. For these experiments, detached leaves from five-week-old tomato plants were inoculated with 5 µL 5×10^5^ per mL spore suspension and disease development was analyzed 3 days after inoculation (DAI). As measured by the size of necrotic lesions, whereas *spr8* plants were more susceptible than WT plants to *B. cinerea* infection, *OE-5* plants were more resistant than WT plants to this pathogen (Figure 7I and 7J). In another pathogen infection assay, 16-day-old seedlings were inoculated *in planta* with spore suspensions of *B. cinerea* and the expression levels of the pathogenesis-related (PR) gene *PR1b1* [59] was examined with qRT-PCR. As shown in Figure 7K, whereas *B.cinerea*-induced expression levels of *PR1b1* were reduced in *spr8* plants than those in WT plants, expression levels of *PR1b1* were much higher in *OE-5* plants than those in WT plants, suggesting that the resistance of plants to pathogen is correlated with the expression levels of defense-related genes.

## Discussion

### *TomLoxD* Is Required for Wound-Induced JA Biosynthesis

Here, we provide several lines of evidence demonstrating that the wound response defect of the tomato *spr8* mutant results from a mutation in *TomLoxD* that is required for wound-induced JA biosynthesis. First, positional cloning studies reveal that *spr8* plants harbor a dominant negative mutation in TomLoxD, a 13-lipoxygenase that catalyzes the oxygenation of the polyunsaturated fatty acid linolenic acid, which is the metabolic precursor of JA. Second, *spr8* leaves accumulate very little JA in response to wounding. The deficiency in wound-induced JA biosynthesis accounts for the defective wound-induced *PIs* expression in *spr8* plants and is consistent with the fact that the wound response phenotype of the mutant can be rescued by exogenous JA. These results lead us to conclude that TomLoxD is responsible for the majority of wound-induced JA production in tomato leaves.

It is worth to note that the *spr8* mutation affects a highly conserved Pro residue (Pro^598^) in the lipoxygenase domain of TomLoxD (Figure S4). As an α-amino acid, Pro contains a distinct cyclic structure and therefore this amino acid exhibits an exceptional conformational rigidity compared to other amino acids [48]. In this context, it is reasonable to speculate that the *spr8* mutation affects the formation of the secondary structure of the TomLoxD protein and hence impairs its activity. Indeed, our data support that, even though the expression of *TomLoxD^P598L^* is still responsive to mechanical wounding (Figure 5A), this mutant version of TomLoxD impairs wound-induced JA biosynthesis (Figure 5B). Considering that the *spr8* mutation occurs in the C-terminal α-helices domain of TomLoxD (Figure 4D), it is most likely that, in *spr8* plants, the TomLoxD^P598L^ protein still can bind the substrate (i.e., linoleic acid) as the WT TomLoxD does, but this mutant protein loses its catalytic activity. Competition between TomLoxD and TomLoxD^P598L^ for substrate binding could underlie that *spr8* acts genetically as a semi-dominant mutant.

As in other higher plants, in tomato lipoxygenases are encoded by a gene family consisting of 6 members (Figure 4E). It has been shown that *TomLoxA*, *TomLoxB*, *TomLoxC* and *TomLoxE* are mainly expressed in fruits during development and ripening [60]. Among them, *TomLoxC* is specifically involved in the generation of C6 aldehydes and alcohols, which are important constituents of volatile flavor of tomato fruits [61]–[63]. The expression of *TomLoxD* and *TomLoxF* is stimulated by the non-pathogenic rhizobacteria *Pseudomonas putida* BTP1 and these two genes are likely to be involved in rhizobacteria mediated-induced systemic resistance [64]. The deduced amino acid sequence of TomLoxD show high similarity to several chloroplast-localized lipoxygenases in *Arabidopsis* that have been shown to be involved in JA biosynthesis. Among them, LOX3 and LOX4 are involved in male fertility [49], [65] whereas LOX2 is specifically involved wound response [66], [67]. TomLoxD also shows high sequence similarity to the maize TASSELSEED1 (TS1) protein, which also encodes a plastid-localized lipoxygenase and plays a critical role in flower development and sex determination [68]. Here, we show that the tomato *TomLoxD* gene is specifically involved in the wound response, but shows minor, if any, effect on general plant growth and flower development (Figure S1). Taken together, these studies indicate that individual lipoxygenase isoforms are differentially regulated and have distinct physiological functions.

### Transgenic Manipulation of *TomLoxD* Leads to Enhanced Resistance of Tomato to Insect and Pathogen Attack

Over two decades ago, Ryan and colleagues discovered the role of JAs in regulating defense gene expression in tomato [1], [20], [22]. Since then an ever growing body of evidence supports the view that the intracellular levels of JA plays a major role in controlling the strength of JA responses. Genetic engineering of plant cells for elevated endogenous JA levels therefore provides a strategy for increasing JA-dependent defenses. Indeed, the Ryan group showed that *35S::PS* plants contain elevated JA levels and constantly express a spectrum of defense-related proteins that provide protection against insect attack [15], [69], [70]. Other examples of genetic alterations that cause constitutive JA accumulation include overexpression of a mitogen-activated protein kinase in tobacco [71] and mutation of the cellulose synthase CeSA3 in *Arabidopsis* [72], [73]. It is noteworthy that even though genetic engineering of the tomato *PS* gene or the *Arabidopsis CeSA3* gene leads to increased JA-dependent resistance against insects or pathogens, the resulting transgenic plants show growth retardation and other physiological defects in normal growth conditions [15], [72], [73], which may limit the application potential of these genes in crop protection.

Attempts to increase endogenous JA levels and thus JA-dependent resistance by overexpression of individual JA biosynthetic genes in tomato and other plants have met with limited success [18], [33], [74], a plausible explanation is that the JA levels are mainly controlled by substrate availability [47], [75], [76]. In contrast to these unsuccessful examples, we show here that *TomLoxD-OE* plants exhibited increased expression levels of wound-induced defense-related genes and are more resistant to *H. armigera*. *TomLoxD-OE* plants also displayed enhanced resistance to the necrotrophic pathogen *B. cinerea*. These results indicated that genetic manipulation of *TomLoxD* leads to enhanced resistance of tomato plants to arthropod herbivores and microbial pathogens.

It is important to note that in the absence of insect attack or pathogen infection, the overall growth and fertility of *TomLoxD-OE* plants were essentially comparable with those of WT plants (Figure S1), indicating there was no fitness cost associated with overexpressing *TomLoxD* in our growth conditions. This is important because the maintenance of constitutive proteins or the continuous mounting of defenses often has severe impacts on plant growth or fertility [77]. Because the overexpression of *TomLoxD* does not impose a significant fitness cost to the plant, the *TomLoxD-OE* plants are viable candidates for field trials to improve insect and pathogen resistance in crop tomato.

Enhanced expression of defense-related genes in *TomLoxD-OE* plants is only observed after mechanical wounding, insect attack or pathogen infection suggests that the activation of the TomLoxD activity is regulated by the JA signaling. Indeed, we found that the wound-induced expression of *TomLoxD* is under the direct regulation of SlMYC2, the functional homolog of the *Arabidopsis* MYC2, a master regulator of JA-responsive gene expression. These findings are consistent with the long-standing observations that JA-signaling and synthesis form an apparent positive feedback regulatory loop [25], [26], [78]. It is also possible that the activity of TomLoxD for wound-induced JA biosynthesis is under posttranscriptional modification and that this modification is regulated by environmental stimuli including wounding, insect attack or pathogen infection. Alternatively, these environmental stimuli could lead to the accumulation of more substrates available for TomLoxD. Given the application potential of TomLoxD for crop protection, it is of significant in future studies to further explore the functional mechanisms of TomLoxD in wound-induced JA biosynthesis.

## Materials and Methods

### Plant Materials and Growth Conditions

Tomato (*Solanum lycopersicum* L.) cv Castlemart (CM) was used as the wild-type (WT) for all experiments. The plant material *35S::PS* used in this study was previously described [15], [17], [29]. Tomato seedlings were grown in growth chambers and maintained under 16 h of light (200 µE m^−2^ s^−1^) at 28°C and 8 h of dark at 18°C and 60% relative humidity.

### Mutant Isolation and Genetic Analysis

Mutagenesis of *35S::PS* plants with ethyl methanesulfonate (EMS) and the isolation of *suppressor of prosystemin-mediated responses* (*spr*) mutants were performed as previously described [17], [29]. *spr8* is one of the identified mutant lines and is deficient in both PPO activity and PI-II protein accumulation.

The original *spr8* mutant in the *35S::PS* genetic background was backcrossed to tomato cv CM as previously described [18]. The identified homozygous *spr8/spr8* mutant plants were crossed to the WT and F_1_ plants were allowed to self-pollinate. The wound response phenotype of F_1_ and F_2_ plants was assessed by measuring PI-II accumulation following wounding treatment.

### Map-Based Clone of *Spr8*

Map-based cloning procedures similar to those described [18], [79] were used to identify the *Spr8* locus. A homozygous *spr8* plant (*S. lycopersicum*) was crossed to the wild tomato species *S. pennellii* (LA716), and the resulting F_1_ plant was backcrossed to the *spr8* parental line to generate a BC_1_ mapping population. The wound-response phenotype of individual BC_1_ plants was scored by measuring PI-II protein levels in response to mechanical wounding, as described above.

Using the BC_1_ population described above, bulked segregant analysis was used in combination with simple sequence repeats (SSR) analysis to identify molecular markers linked to *Spr8*. Equal amounts of genomic DNA from10 randomly selected wound-responsive (i.e., wild-type) and 10 nonresponsive (i.e., mutant) BC_1_ plants were pooled to construct a wild-type DNA bulk (B^+^) and a mutant DNA bulk (B^−^), respectively. Rough mapping using the 20 BC_1_ plants indicated that the target gene is linked to the marker TES0023 on the long arm of chromosome 3. Analysis of linkage between *Spr8* and known SSR markers in this region demonstrated that *Spr8* is located between TES0023 and TES1203. A high-resolution genetic map of the *Spr8* region was constructed by scoring 354 BC_1_ plants for recombination events within the SSR601-*Spr8*-M140 interval in the scaffold SL2.40sc03701 of the sequenced tomato genome. Sequence analyses of genes in this interval revealed a C-to-T mutation in the *TomLoxD* gene. DNA primers for molecular markers used in map-based cloning were listed in Table S1.

For complementation analysis, the *35S_pro_:TomLoxD-GFP* construct was introduced into the *spr8* plants using *Agrobacterium tumefaciens*-mediated transformation for the complementation analysis. The *TomLoxD-RNAi* and *35S_pro_:TomLoxD^P598L^-GFP* constructs were introduced into WT plants using *Agrobacterium tumefaciens*-mediated transformation.

### DNA Constructs and Plant Transformation

DNA constructs for plant transformation were generated following standard molecular biology protocols and Gateway (Invitrogen) technology. Full-length coding sequence of *TomLoxD* was amplified with Gateway-compatible primers. The PCR product was cloned by pENTR Directional TOPO cloning kits (Invitrogen) and then recombined with the binary vector pGWB5 (35S promoter, *C-GFP*) to generate the *35S_pro_:TomLoxD-GFP* construct. Similarly, we generated *35S_pro_:TomLoxD^P598L^-GFP* construct, which was amplified from *spr8* cDNAs, using the same primers as *35S_pro_:TomLoxD-GFP* construct. Full-length coding sequence of *SlMYC2* was also cloned into the pGWB17 vector (35S promoter, C-4myc) to generate the *35S_pro_:SlMYC2-4myc* constructs.

To generate a *TomLoxD-RNAi* construct, fragments of the *TomLoxD* open read frame (106–570 bp), which were amplified from the cDNAs, were digested by *Xho*I and *Spe*I, and then inserted into *Xho*I-*Spe*I sites and *Sal*I-*Xba*I sites in PUCCRNAi vector by steps. So this second ligation inserts the PCR product was in inverted orientation with respect to first cloned fragment, yielding an inverted repeat separated by the first intron fragment of *GA20 oxidase* from potato. The two reversed repeated DNAs were transferred to pCAMBIA-1301 (CAMBIA) from PUCCRNAi by *Pst*I digestion. The plasmid pCAMBIA-1301 had been modified by adding a CaMV 35S promoter. Similarly, the *SlMYC2-RNAi* construct was performed. All primers used for DNA construct generation are listed in Table S3 online.

The above constructs were then transformed into *Agrobacterium tumefaciens* strain AGLO and used to transform tomato cotyledon explants as described previously [18]. Transformants were selected based on their resistance to hygromycin. Homozygous T_3_ or T_4_ transgenic seedlings were used for phenotype and molecular characterization.

### PI-II Protein Accumulation Assays

The wound response of tomato plants was determined using a radial immunodiffusion assay for the detection of PI-II accumulation in leaf tissue as previously described [11], [36]. Two-leaf-stage (16-day-old) seedlings were used for the wounding treatment as described [29] and then the wounded leaf (local response) and the unwounded leaf (systemic response) were harvested separately to assay PI-II protein level.

### Wounding, Systemin and MeJA Treatment of Tomato Plants

For wounding treatment, 16-day-old seedlings were wounded with a hemostat across the midrib of all leaflets on the lower leaf and the upper leaf. Then, the same leaflets were wounded again, proximal to the petiole. Wounded plants were incubated under continuous illumination conditions. For each time point of sampling, five whole plants leaves were harvested for the extraction of RNAs.

Systemin feeding experiments were performed using 16-day-old tomato seedlings as previously described with minor modifications [18], [28], [29]. Briefly, 2.5 pmol systemin was diluted from stock solutions into 300 µL 15 mM sodium phosphate, pH 6.5, prior to use. Tomato seedlings were excised at the base of the stem and placed in 0.5 mL microfuge tubes containing 300 µL dilutions. When >90% of the elicitor solution had been imbibed (approximately 2 hours), plants were transferred to glass vials containing 20 mL of water, and incubated in a Lucite Box under continuous light. Twelve hours later, leaf tissues of five plants were pooled for RNA extraction and gene expression assays. Control plants were fed with sodium phosphate buffer. Systemin was commercially synthesized by Shanghai Sangon Biological Engineering & Technology and Service Co. Ltd (Shanghai, PR China).

Sixteen-day-old tomato seedlings were treated with MeJA as described previously [80]. Control plants were incubated in a separate container in which ethanol was applied to cotton wicks. Twelve hours later, leaf tissues of five plants were pooled for RNA extraction. MeJA was purchased from Sigma-Aldrich.

### Gene Expression Analysis

For qRT-PCR analysis, leaf tissues were harvested and frozen in liquid nitrogen for RNA extraction. RNA extraction and qRT-PCR analysis were performed as previously described [50]. Expression levels of target genes were normalized to those of the tomato *Actin2* gene. Primers used to quantify gene expression levels are listed in Table S2.

### Analysis of Trichomes

To examine the general pattern of trichome distribution on the adaxial surface of leaves, small pieces of tissue (5×5 mm), on the same base region of the third leaves from bottom to upper, were fixed, dehydrated, critical point dried in CO_2_, and coated with a film of gold as described [81]. Observations were performed with a HITACHI S-3000N scanning electron microscope (Japan) at an accelerating voltage of 15 kV. The density of type VI trichomes on the adaxial surface of leaves was determined by counting trichomes with a dissecting microscope equipped with a stage micrometer. All measurements were performed on WT and *spr8* plants grown side by side under the same growth conditions.

Five-week-old plants were used to isolated type VI trichomes of leaves to obtain trichome exudates as previously described with minor modified [43]. Briefly, 1, 000 heads of Type VI glandular trichomes were selectively collected with a stretched-glass pipette and dissolved into 200 µL methyl tert-butyl ether (MTBE, Sigma) to analysis the chemical structures of compounds by GC-MS as described [43]. Different concentrations of external standards were run under the same GC conditions to develop standard curves to quantify volatiles (2-carene for monoterpenes, β-caryophyllene for sesquiterpenes).

### Insect Feeding Trials

General procedures for rearing and handling cotton bollworm (*Helicoverpa armigera*) were described previously [18], [79]. The average larval weight at the beginning of the feeding trial was ∼5 mg. After termination of the feeding trial, PI-II protein accumulation in the remaining leaf tissues was measured [11], [36], as was the weight gain of larvae reared on both of the host genotypes.

### Plant Infection with *Botrytis cinerea*

Detached leaves of five-week-old plants were inoculated as previously described [51]. For qRT-PCR experiments, the inoculation tests were performed *in planta* as described [58]. The same experiment was done with mock-pretreated plants as control. After inoculated for different times, the samples were then harvested for RNA extraction.

### Sequence Analysis

The BLAST search program [82] was used for sequence analysis. The software ClusterX and T-coffee (http://www.ebi.ac.uk/Tools/t-coffee/) were used for sequence alignment. The phylogenetic relationship of TomLoxD in plants is inferred from protein sequences using a Bayesian approach in MrBayes [83]. The node labels are measures of support, which indicate the proportion of trees in the posterior distribution to containing the node.

### JA Quantification

For JA content measurement, 16- to 18-day-old plant leaves were wounded as described above. Approximately 200 mg leaf tissue (fresh weight) from five different plants was pooled for JA quantification as described previously [84]. Leaf tissues were also harvested from unwounded plants as controls.

### ChIP-PCR Assays

ChIP assays were performed following a published protocol [50], [51], [54], [85] with minor modifications. Briefly, 1 hour after wounding treatment, 2.0 gram of 16-day-old *35S_pro_:SlMYC2-4myc* plant leaves were harvested and cross-linked in 1% formaldehyde for ChIP experiment. myc antibody (Millipore) was used to immunoprecipitate the protein-DNA complex, and the precipitated DNA was purified using a PCR purification kit (Qiagen) for PCR analysis. Chromatin precipitated without antibody was used as negative control, while the isolated chromatin before precipitation was used as input control. Primers used for ChIP-PCR are listed in Table S4 online.

### Electrophoretic Mobility Shift Assay

For plasmid construction of maltose binding protein (MBP) fusions with SlMYC2, the cDNA was amplified and cloned into the pMAL-c2 vector (New England Biolabs, Beverly, MA) via *Bam*HI and *Pst*I restriction sites. The MBP-SlMYC2 recombinant protein was expressed in the BL21 *Escheichia coli* (*E. coli*) strain and purified by binding onto an amylose resin (New England Biolabs) column, according to the instructions provided by the manufacturer. The 50-bp *TomLoxD* promoter probes containing G-box-like motif at the -369 site were synthesized and labeled with biotin at the 3′ end (Invitrogen), which containing the same sequences as that of the competitor probes without biotin-labled, while the mutated labeled probes were deleted the G-box-like motif. EMSA assays were performed using a LightShift Chemiluminescent EMSA kit (Thermo Scientific) as described [54]. Probe sequences are shown in Table S4 online.

### Transient Expression Assay in *N. benthamiana* Leaves

The transient expression assays were performed in *N. benthamiana* leaves as previously described [51], [54]. The *TomLoxD* promoter was amplified and cloned into the pCAMBIA1381-Z (CAMBIA) via *Eco*RI and *Pst*I restriction sites to generate the reporter construct *TomLoxD_pro_:LUC*. The *SlMYC2* effector construct was the above-described *35S_pro_:SlMYC2-4myc*. We used a low-light cooled CCD imaging apparatus (NightOWL II LB983 with indigo software) to capture the LUC image and to count luminescence intensity. The leaves were sprayed with 100 mM luciferin and were placed in darkness for 3 min before luminescence detection.

### Transient Expression Assay in *Arabidopsis* Protoplast Cells

For plasmid construction of *35S_pro_:TomLoxD-GFP*, the full length cDNA was amplified and cloned into the pGFP-2 vector [86] via *Xho*I and *Kpn*I restriction sites to generate *35S_pro_:TomLoxD-GFP*. Protoplast isolation and analysis of the subcellular location of transiently expressed GFP fusions by confocal fluorescence microscopy were performed as described [87].

### Pollen Viability Assays

Alexander's triple staining was employed to measure viability of pollens, which were freshly harvested, as described previously [88].

### Accession Numbers

The accession number of the sequenced tomato genome for the scaffold containing the *Spr8/TomLoxD* gene is SL2.40sc03701. The accession number from SGN database as following: *TomLoxD* (*Solyc03g122340*); SlMYC2(Solyc08g076930). Sequence data from this article can be found in the in the *Arabidopsis* Genome or GenBank databases under accession number as following: *Arabidopsis thaliana* AtLOX1 (AT1G55020), AtLOX2 (AT3G45140), AtLOX3 (AT1G17420), AtLOX4 (AT1G72520), AtLOX5 (AT3G22400), AtLOX6 (AT1G67560); *Solanum lycopersicum* TomLoxA (P38415), TomLoxB (P38416), TomLoxC (AAB65766), TomLoxD (AAB65767), TomLoxE (AAG21691), TomLoxF (NP_001234259); Zea mays ZmTS1 (ACL81190); *Solanum tuberosum* LOXH3 (CAA65269), StLOXH1 (CAA65268), STLOX (AAD09202), POTLX-3 (AAB67865), St13s-LOX2-1 (O24370), St13s-LOX3-1 (O24371); *Nicotiana tabacum* NtLOX (CAA58859); *Glycine max* Gm13-LOX3-1 (XP_003528556); *Oryza sativa* Japonica Group OsLOX6 (NP_001049158); *Rattus norvegicus* RnLOX3 (NP_001099263); *Mus musculus* Mm5-LOX (NP_033792); *Homo sapiens* HsLOX3 (CAC12843).

## Supporting Information

Figure S1 Growth and reproductive phenotypes of *spr8* and *TomLoxD-OE* plants. (A) Photographs of the overall growth rate and morphology from WT (left), *spr8* (middle) and *OE-5* (right). (B) Flowers of WT (left), *spr8* (middle) and *OE-5* (right). (C) Alexander's triple staining showing viable (red) pollen from WT (left), *spr8* (middle) and *OE-5* (right) anthers.
(TIF)

Figure S2 Time-course expression of the wound-induced genes *TD* (A) and *LapA* (B) in WT and *spr8* plants. Sixteen-day-old seedlings of WT (black bar) and *spr8* (white bar) plants containing two fully expanded leaves were mechanically wounded with a hemostat on both leaves. At indicated times (hours) after wounding, leaf tissues were harvested for RNA extraction and qRT-PCR assays. Data presented are mean values of three biological repeats with SD.
(TIF)

Figure S3 Wound response of F_1_ plants between WT and the *spr8* mutant. (A–C) qRT-PCR analysis of wound-induced expression of *PI-II* (A), *TD* (B) and *LapA* (C) in WT, (WT×*spr8*) F_1_ (F_1_) and *spr8* plants as shown. Sixteen-day-old plants were mechanically wounded with a hemostat at the distal end of each leaflet. Twelve hours after wounding, wounded leaves (black bar) were harvested for quantification transcript levels. Unwounded leaves (white bar) were used as control. Data presented are mean values of three biological repeats with SD.
(TIF)

Figure S4 Multiple sequence alignment of TomLoxD and related lipoxygenases from different plant species. Sequences were aligned with DNAMAN. The five-pointed star indicates the Pro residue which was mutated to an Leu in *spr8* plants.
(TIF)

Figure S5 Wound response of *TomLoxD-RNAi*, *TomLoxD^P598L^-OE*/WT and *TomLoxD-OE*/*spr8* plants. (A) and (B) Wound-induced expression of *TomLoxD* (A) and *PI-II* (B) in *TomLoxD-RNAi* and *TomLoxD^P598L^-OE* plants. (C) and (D) Wound-induced expression of *TomLoxD* (C) and *PI-II* (D) in *spr8* and *TomLoxD-OE*/*spr8* plants. Sixteen-day-old plants containing two fully expanded leaves were wounded with a hemostat on both leaves. One hour (A, C) or 12 hours (B, D) after wounding, wounded leaves were harvested for RNA extraction and qRT-PCR assays (black bar). Gene expression in leaves of unwounded plants (white bar) served as control. Data shown are mean ± SD of three independent assays.
(TIF)

Figure S6 Subcellular localization of TomLoxD in *Arabidopsis* leaf protoplast cells. (A) Fluorescence of 35S:TomLoxD-GFP. (B) Chloroplast auto fluorescence. (C) Bright-field images of a mesophyll cell protoplast of *Arabidopsis*; (D) Merge image of (A) and (B).
(TIF)

Figure S7 Wound-induced expression of *PI-II* in *SlMYC2-RNAi* plants. Sixteen-day-old WT and *SlMYC2-RNAi* plants containing two fully expanded leaves were wounded with a hemostat on both leaves. Twelve hours after wounding, leaf tissues from six wounded plants were harvested for RNA extraction and qRT-PCR analysis of *PI-II* expression (black bar, W). *PI-II* expression in leaves of unwounded plants (white bar, U) served as a control.
(TIF)

Table S1 DNA primer pairs used for map-based cloning and diagnostic PCR.
(XLSX)

Table S2 DNA primer pairs used for qRT-PCR.
(XLSX)

Table S3 DNA primer pairs used for constructs generation.
(XLSX)

Table S4 DNA primer pairs used for EMSA and ChIP-PCR assays.
(XLSX)
