# Supplementary figures and images for: Role of Tomato Lipoxygenase D in Wound-Induced Jasmonate Biosynthesis and Plant Immunity to Insect Herbivores
Source: PLoS Genet. 2013 Dec 12;9(12):e1003964. doi: 10.1371/journal.pgen.1003964 (PMC3861047; doi:10.1371/journal.pgen.1003964)

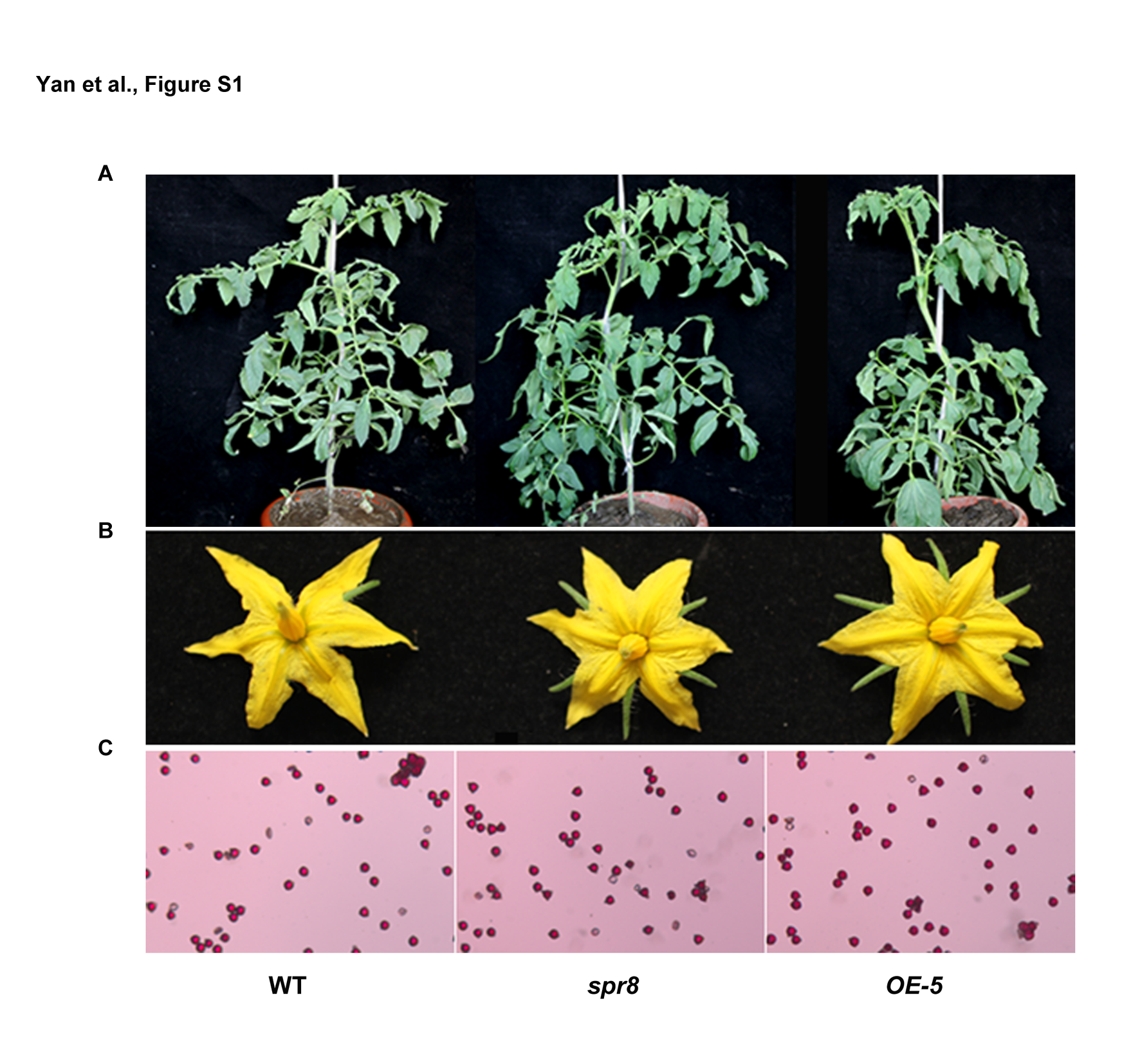

Supplement: Figure S1 — Growth and reproductive phenotypes of spr8 and TomLoxD-OE plants. (A) Photographs of the overall growth rate and morphology from WT (left), spr8 (middle) and OE-5 (right). (B) Flowers of WT (left), spr8 (middle) and OE-5 (right). (C) Alexander's triple staining showing viable (red) pollen from WT (left), spr8 (middle) and OE-5 (right) anthers. (TIF) [file pgen.1003964.s001.tif]

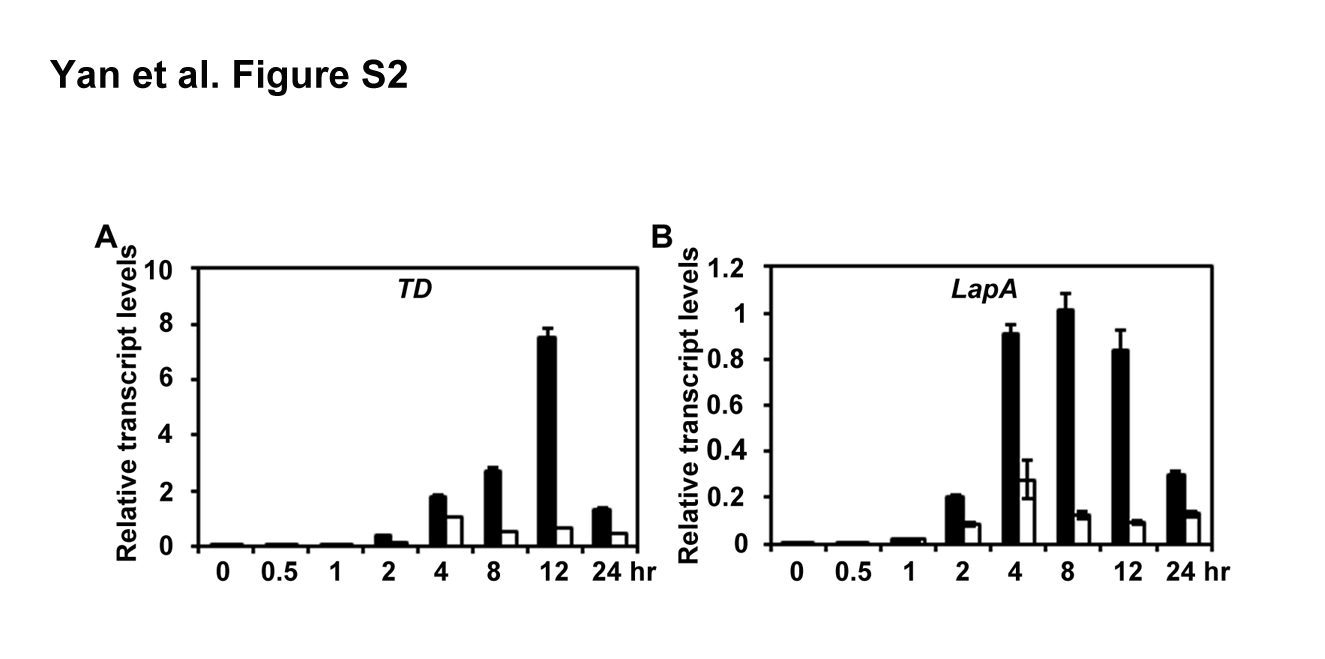

Supplement: Figure S2 — Time-course expression of the wound-induced genes TD (A) and LapA (B) in WT and spr8 plants. Sixteen-day-old seedlings of WT (black bar) and spr8 (white bar) plants containing two fully expanded leaves were mechanically wounded with a hemostat on both leaves. At indicated times (hours) after wounding, leaf tissues were harvested for RNA extraction and qRT-PCR assays. Data presented are mean values of three biological repeats with SD. (TIF) [file pgen.1003964.s002.tif]

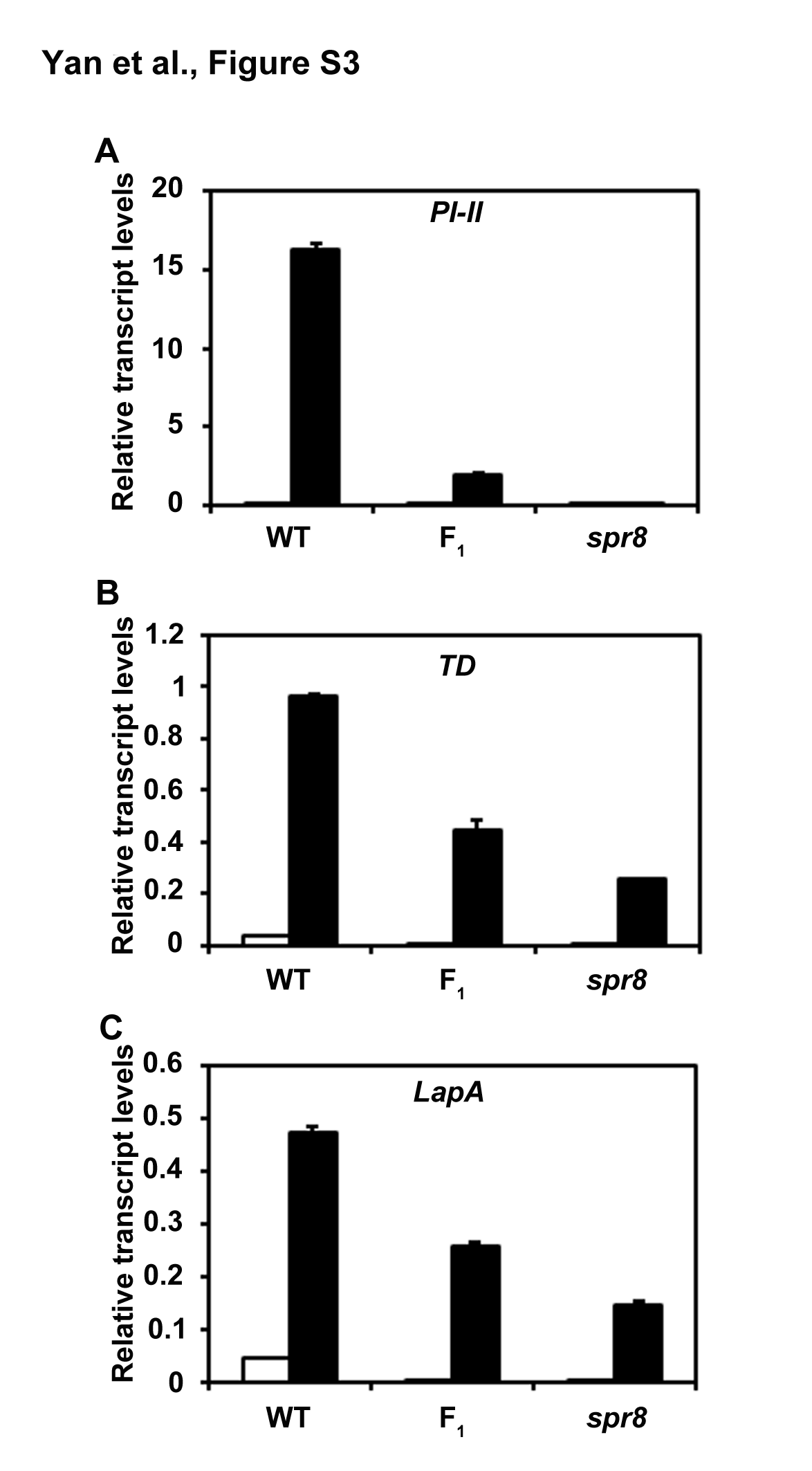

Supplement: Figure S3 — Wound response of F1 plants between WT and the spr8 mutant. (A–C) qRT-PCR analysis of wound-induced expression of PI-II (A), TD (B) and LapA (C) in WT, (WT×spr8) F1 (F1) and spr8 plants as shown. Sixteen-day-old plants were mechanically wounded with a hemostat at the distal end of each leaflet. Twelve hours after wounding, wounded leaves (black bar) were harvested for quantification transcript levels. Unwounded leaves (white bar) were used as control. Data presented are mean values of three biological repeats with SD. (TIF) [file pgen.1003964.s003.tif]

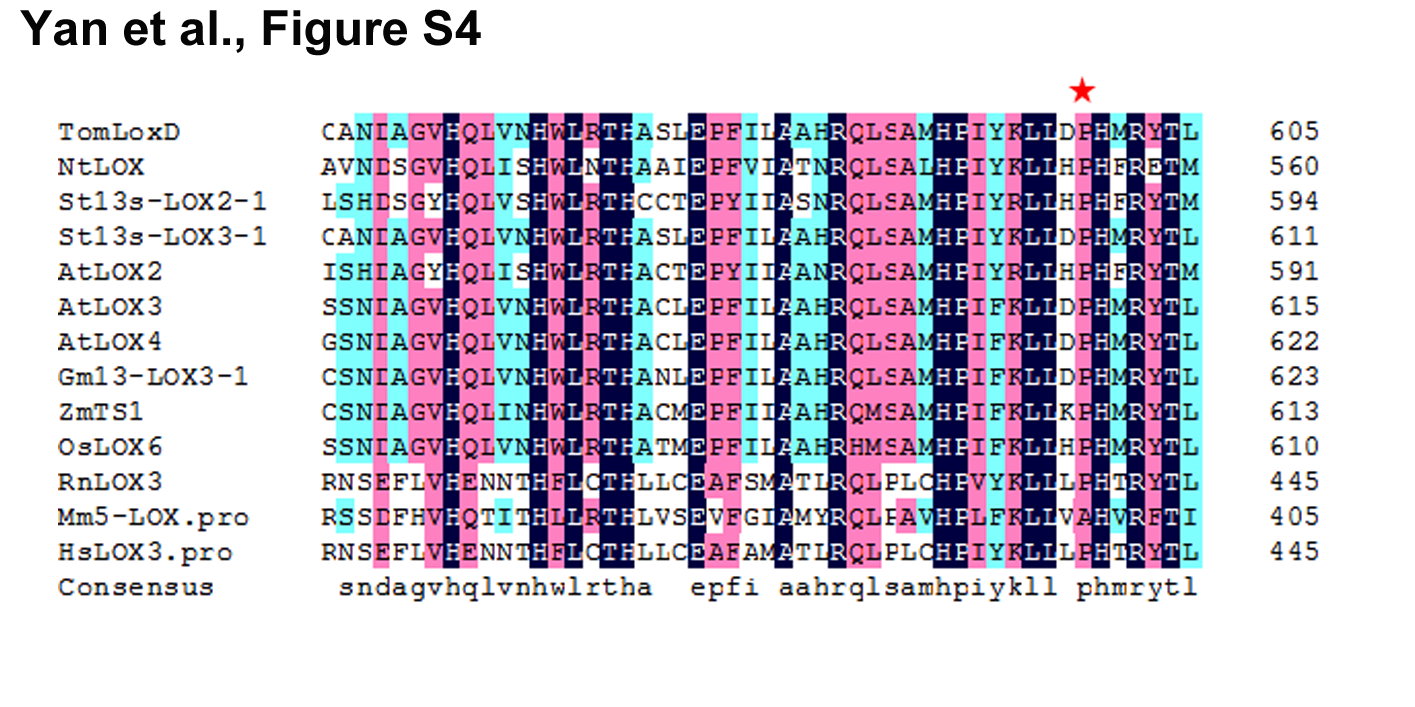

Supplement: Figure S4 — Multiple sequence alignment of TomLoxD and related lipoxygenases from different plant species. Sequences were aligned with DNAMAN. The five-pointed star indicates the Pro residue which was mutated to an Leu in spr8 plants. (TIF) [file pgen.1003964.s004.tif]

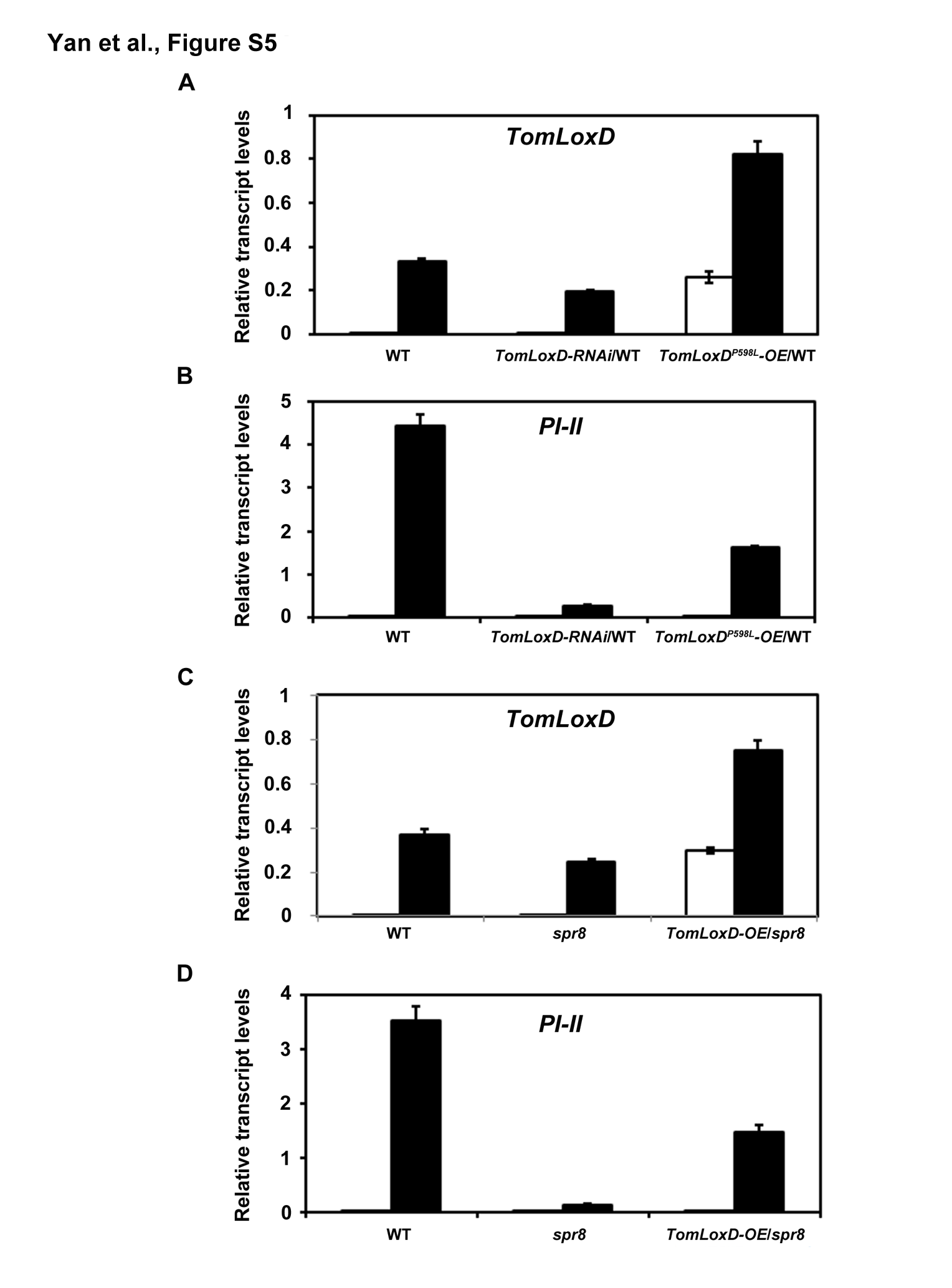

Supplement: Figure S5 — Wound response of TomLoxD-RNAi, TomLoxDP598L-OE/WT and TomLoxD-OE/spr8 plants. (A) and (B) Wound-induced expression of TomLoxD (A) and PI-II (B) in TomLoxD-RNAi and TomLoxDP598L-OE plants. (C) and (D) Wound-induced expression of TomLoxD (C) and PI-II (D) in spr8 and TomLoxD-OE/spr8 plants. Sixteen-day-old plants containing two fully expanded leaves were wounded with a hemostat on both leaves. One hour (A, C) or 12 hours (B, D) after wounding, wounded leaves were harvested for RNA extraction and qRT-PCR assays (black bar). Gene expression in leaves of unwounded plants (white bar) served as control. Data shown are mean ± SD of three independent assays. (TIF) [file pgen.1003964.s005.tif]

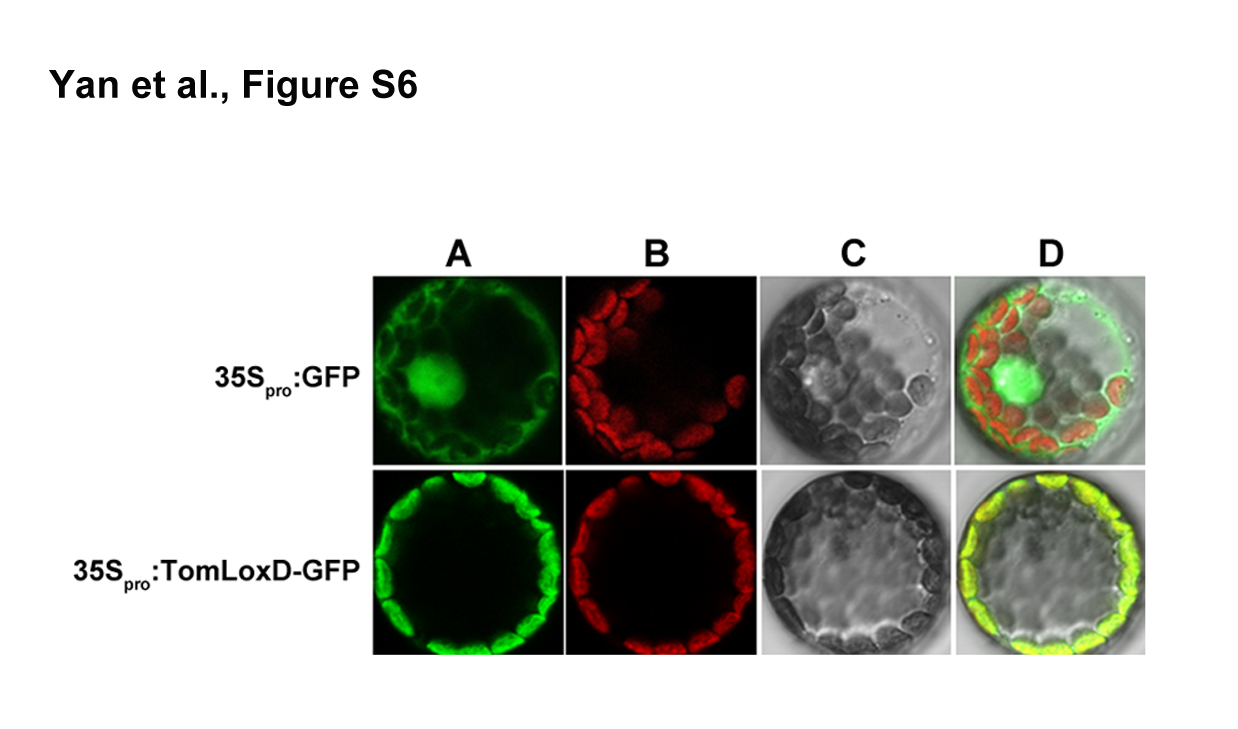

Supplement: Figure S6 — Subcellular localization of TomLoxD in Arabidopsis leaf protoplast cells. (A) Fluorescence of 35S:TomLoxD-GFP. (B) Chloroplast auto fluorescence. (C) Bright-field images of a mesophyll cell protoplast of Arabidopsis; (D) Merge image of (A) and (B). (TIF) [file pgen.1003964.s006.tif]

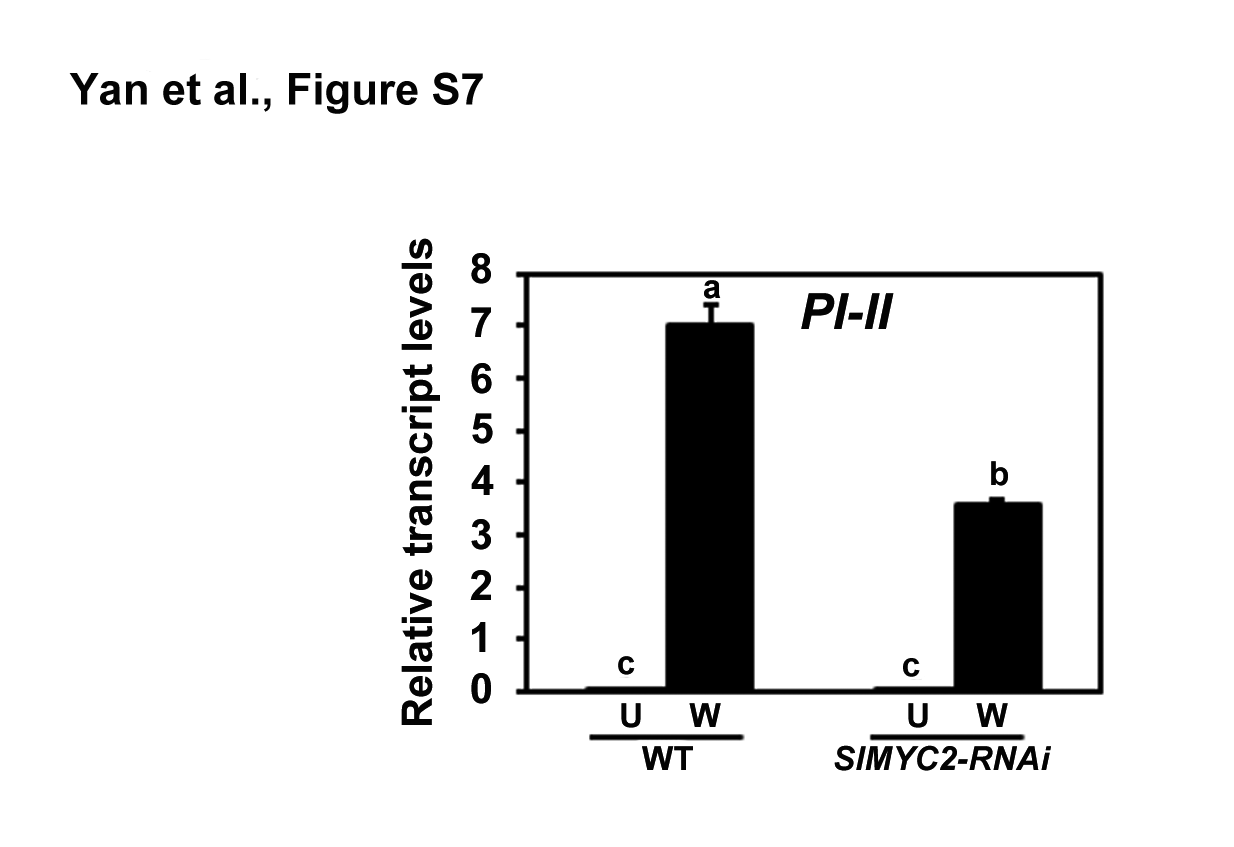

Supplement: Figure S7 — Wound-induced expression of PI-II in SlMYC2-RNAi plants. Sixteen-day-old WT and SlMYC2-RNAi plants containing two fully expanded leaves were wounded with a hemostat on both leaves. Twelve hours after wounding, leaf tissues from six wounded plants were harvested for RNA extraction and qRT-PCR analysis of PI-II expression (black bar, W). PI-II expression in leaves of unwounded plants (white bar, U) served as a control. (TIF) [file pgen.1003964.s007.tif]
